# Enhancement of Breast Cancer Cell Aggressiveness by lncRNA *H19* and its Mir-675 Derivative: Insight into Shared and Different Actions

**DOI:** 10.3390/cancers12071730

**Published:** 2020-06-29

**Authors:** Evodie Peperstraete, Clément Lecerf, Jordan Collette, Constance Vennin, Ludivine Raby, Pamela Völkel, Pierre-Olivier Angrand, Marie Winter, François Bertucci, Pascal Finetti, Chann Lagadec, Samuel Meignan, Roland P. Bourette, Xuefen Le Bourhis, Eric Adriaenssens

**Affiliations:** 1 University Lille, CNRS, INSERM, CHU Lille, Centre Oscar Lambret, UMR 9020–UMR 1277–Canther–Cancer Heterogeneity, Plasticity and Resistance to Therapies, F-59000 Lille, France; evodie.peperstraete.etu@univ-lille.fr (E.P.); clement.lecerf@univ-lille.fr (C.L.); jorkan62@gmail.com (J.C.); constance.vennin@gmail.com (C.V.); ludivine.raby@gmail.com (L.R.); pamela.voelkel@univ-lille.fr (P.V.); pierre-olivier.angrand@univ-lille.fr (P.-O.A.); marie.winter.etu@univ-lille.fr (M.W.); chann.lagadec@inserm.fr (C.L.); samuel.meignan@inserm.fr (S.M.); Roland.BOURETTE@ibl.cnrs.fr (R.P.B.); xuefen.le-bourhis@univ-lille.fr (X.L.B.); 2 Laboratoire d’Oncologie Prédictive, CRCM, Institut Paoli-Calmettes, INSERM UMR1068, CNRS UMR7258, Aix-Marseille Université, Département d’Oncologie Médicale, Institut Paoli-Calmettes, 13009 Marseille, France; BERTUCCIF@ipc.unicancer.fr (F.B.); FINETTIP@ipc.unicancer.fr (P.F.); 3 Tumorigenesis and Resistance to Treatment Unit, Centre Oscar Lambret, F-59000 Lille, France

**Keywords:** LncRNA, *H19* gene, breast cancer, miR-675, cancer stem cell, tumoral progression

## Abstract

Breast cancer is a major public health problem and the leading world cause of women death by cancer. Both the recurrence and mortality of breast cancer are mainly caused by the formation of metastasis. The long non-coding RNA *H19*, the precursor of miR-675, is involved in breast cancer development. The aim of this work was to determine the implication but, also, the relative contribution of *H19* and miR-675 to the enhancement of breast cancer metastatic potential. We showed that both *H19* and miR-675 increase the invasive capacities of breast cancer cells in xenografted transgenic zebrafish models. In vitro, *H19* and miR-675 enhance the cell migration and invasion, as well as colony formation. *H19* seems to induce the epithelial-to-mesenchymal transition (EMT), with a decreased expression of epithelial markers and an increased expression of mesenchymal markers. Interestingly, miR-675 simultaneously increases the expression of both epithelial and mesenchymal markers, suggesting the induction of a hybrid phenotype or mesenchymal-to-epithelial transition (MET). Finally, we demonstrated for the first time that miR-675, like its precursor *H19,* increases the stemness properties of breast cancer cells. Altogether, our data suggest that *H19* and miR-675 could enhance the aggressiveness of breast cancer cells through both common and different mechanisms.

## 1. Introduction

Long non-coding RNAs (lncRNAs) (>200 nt) are essential in cell biology, and their dysfunction plays a critical role in cancer development and progression. Indeed, lncRNAs are involved in diverse cellular processes such as cell proliferation, apoptosis, differentiation and pluripotency, but their mechanisms of action remain largely undeciphered [1]. Among these lncRNAs, *H19*, the first lncRNA discovered located in the *H19*/*IGF2* locus, is a subject of interest. 

*H19* is submitted to genomic imprinting [2] and is expressed during embryonic development. Its expression is repressed after birth, except in a few tissues like the mammary gland, renal gland and uterus [3,4,5,6]. Many studies have shown that *H19* promotes tumor phenotypes and induces metastasis in various cancers like gastric, colorectal, bladder, renal, lung and breast cancers but, also, in glioblastoma [7,8,9,10,11,12,13]. We have previously shown that *H19* is overexpressed in 70% of breast cancer and promotes the tumorigenic properties of cancer cells [3,14]. The *H19* gene is upregulated by transcription factors such as E2F1 to enhance the cell cycle progression and cell invasion [12].

*H19* can exert its protumorigenic function through diverse molecular mechanisms like the targeting of transcriptional factors or chromatin modifier complexes such as PRC2 (polycomb repressive complex 2) [1]. *H19* binds and recruits the histone methyltransferase EZH2 to the promoter of the proapoptotic gene *BIK* (BCL-2 interacting killer), inducing a reshaping of the chromatin (by trimethylation of the lysine 27 of histone H3) and an inhibition of the BIK transcription [15]. *H19* also interacts with microRNAs (regulatory small non-coding RNAs) to serve as a sponge by sequestering them and inhibiting their actions. For instance, *H19* sponges miR-let7 to maintain the breast cancer stem cells status [16]. Moreover, *H19* increases the expression of DNMT1, a DNA methyltransferase, by sponging miR-152, thus inducing the growth and invasion of breast cancer cells [17]. 

In addition, *H19* could generate two mature miRNAs, miR-675-5p (miR-675) and miR-675-3p (miR-675*) [18]. These miRNAs mainly act as posttranscriptional repressors by interacting with the mRNA target [19]. *H19*-derived miR-675 favors the tumor progression by repressing several well-known tumor suppressor genes, such as *Rb* [20], *Twist1* [21] or *RUNX1* [22]. We have identified c-Cbl and Cbl-b, two ubiquitin ligase E3, as specific targets of miR-675-5p in breast cancer cells [23].

We have already demonstrated the oncogenic role of the *H19* gene in breast tumorigenesis [14], and *H19*-derived miR-675 has been reported to promote the tumorigenesis of several cancers [20,22,23]. In this study, we examined the involvement and the relative contribution of *H19* and miR-675 in promoting breast cancer cell aggressiveness. Our results indicate that *H19* and miR-675 are in favor of cell migration, invasion and stemness through both common and different mechanisms. 

## 2. Results

### 2.1. LncRNA H19 and miR-675 Promote Breast Cancer Cell Invasion in Zebrafish Xenograft Model

**Figure 1:**
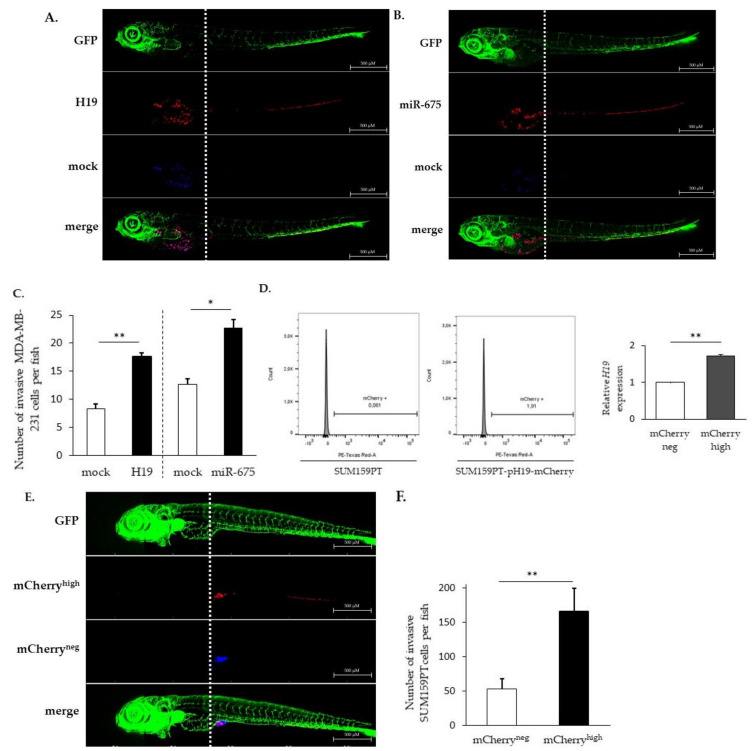
*H19* and miR-675 both promote cancer cell invasion in vivo. (**A**) Invasive capacities of MDA-MB-231 stably overexpressing *H19* and the control, stained with lipophilic tracers, in transgenic zebrafish. Fluorescent pictures were captured using automated image acquisition software. (**B**) Invasive capacities of MDA-MB-231 stably overexpressing miR-675 and the control, stained with lipophilic tracers, in transgenic zebrafish. Fluorescent pictures were captured using automated image acquisition software. (**C**) Quantification of invasive cells per zebrafish. (**D**) mCherry protein fluorescence in SUM159PT transfected or not with pH19-mCherry plasmid. Fluorescence intensity is categorized in mCherry^neg^ and mCherry^high^ cellular subpopulations. Relative *H19* expression in those subpopulations is figured. (**E**) Invasive capacities of mCherry^neg^ and mCherry^high^ cellular subpopulations, stained with lipophilic tracers, in transgenic zebrafish. Fluorescent pictures were captured using automated image acquisition software. (**F**) Quantification of invasive cells per zebrafish. For each experiment, forty embryos were used. *p < 0.05 and **p < 0.01.

A tumor cell transplantation in zebrafish embryos represents a simple and rapid approach to study a tumor cell invasion and metastasis. The optical transparency of the embryos offers the advantage to monitor cancer cell behavior within a few days after the transplantation [24]. In order to investigate the relative contribution of *H19* and miR-675 in the metastatic process in vivo, breast cancer cells, stained with liposoluble fluorophores, were injected into the yolk sac of transparent transgenic zebrafish embryos having their entire vascular system labeled with green fluorescence, and the invasion of the injected cells was evaluated three days post-injection, as described in Materials and Methods. An increased invasion was observed for MDA-MB-231 breast cancer cells stably overexpressing *H19* or miR-675 compared to the control cells (Figure 1A–C). 

In order to highlight the role of the *H19* gene without ectopic overexpression, the SUM159PT breast cancer cell line was transfected with a vector expressing the stable red fluorescent protein mCherry under the control of the *H19* promoter. This reporter system allowed us to select cells overexpressing *H19* within the total population without modulating its expression.

As expected, the mCherry^high^ cell fraction displayed a higher *H19* expression when compared to the mCherry^neg^ cell fraction (Figure 1D), validating the established cellular model. Of note, the level of *H19* in mCherry^high^ cells may vary according to the experiments (2 to 14 times higher than mCherry^neg^ cells). Thus, we were able to isolate cells overexpressing lncRNA *H19* from a heterogeneous cellular population, and we chose a population overexpressing two-fold the *H19* gene to conduct further experiments. 

SUM159PT pH19-mCherry^neg/high^ cells, stained with liposoluble fluorophores, were coinjected into the yolk sac of transgenic zebrafishes. We showed that mCherry^high^ cells move further towards the caudal end through the dorsal aorta, caudal artery and then caudal vein compared to mCherry^neg^ cells (Figure 1E). As shown in Figure 1E,F, mCherry^high^ cells were three times more invasive than mCherry^neg^ cells, further confirming that cells expressing higher levels of *H19* were more invasive.

The H19- and miR-675-enhanced breast cancer cell invasions in vivo prompted us to determine if and how H19 and miR-675 could affect breast cancer cells in vitro in terms of migration, invasion, epithelial-to-mesenchymal transition (EMT) and stemness, as all these processes are known to be involved in tumor invasion.

### 2.2. LncRNA H19 and miR-675 Enhance Breast Cancer Cell Migration and Invasion in Collagen Gel

**Figure 2:**
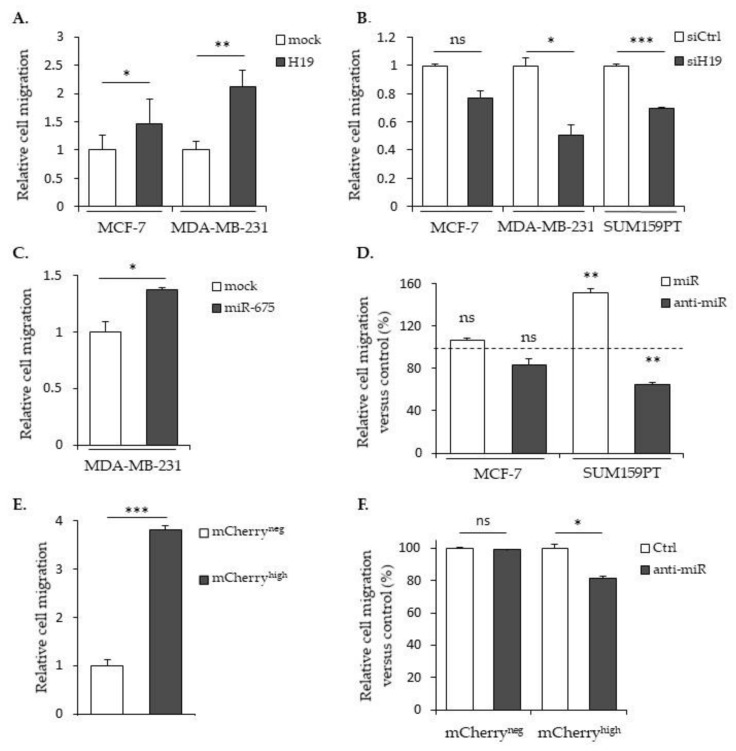

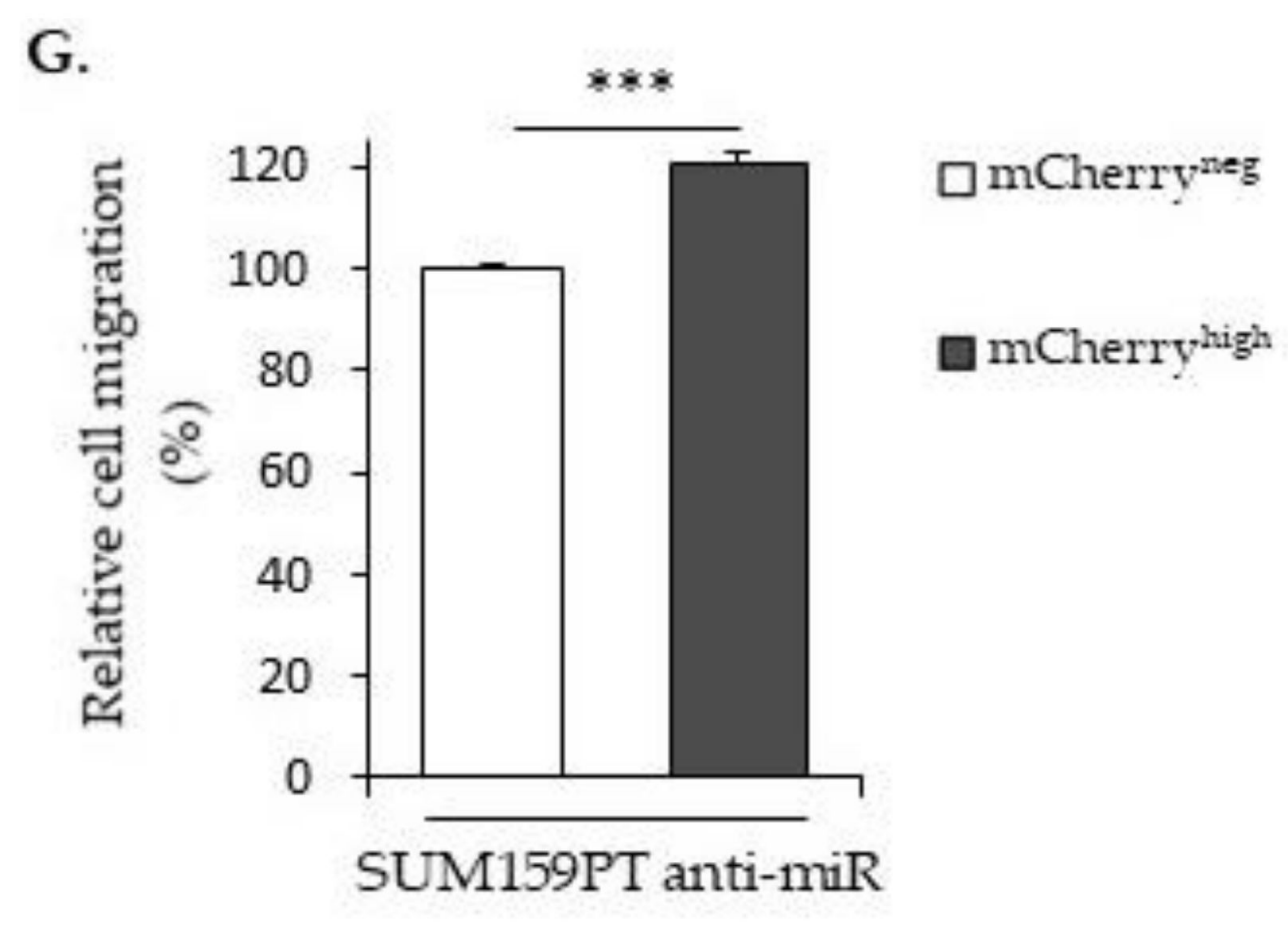
*H19* and miR-675 both promote breast cancer cell migrations. (**A**) Control (mock) or *H19*-stably overexpressing cells (H19) were cultured in Transwell for 24 h. Migrated cells were then incubated with 1-mM Hoescht 33258 and counted. (**B**) Migratory capacities of the control (siCtrl) and *H19*-knockdown cells (siH19) determined by Transwell assay. (**C**) MDA-MB-231 control (mock) or miR-675-stably overexpressing cells (miR-675) were cultured in Transwell for 24 h. Migrated cells were then incubated with 1-mM Hoescht 33258 and counted. (**D**) Migratory capacities of miR-675-transfected cells (miR) or miR-675-specific inhibitor transfected cells (anti-miR) determined by Transwell assay. Results are presented as a percentage of the control. (**E**) Relative migratory capacities of SUM159PT-pH19-mCherry^neg^ versus SUM159PT-pH19-mCherry^high^ determined by Transwell assay. (**F**) Migratory capacities of miR-675-specific inhibitor transfected cells (anti-miR). Results are presented as a percentage of the control. (**G**) Migratory capacities of miR-675-specific inhibitor transfected cells (anti-miR). Results are presented as a percentage of the mCherry^neg^ condition. * *p* < 0.05; ** *p* < 0.01; *** *p* < 0.001; ns: not significant.

The migratory capacities of breast cancer cells overexpressing or not *H19* or miR-675 were determined by using Transwell assays. In MCF-7 or MDA-MB-231 cells stably overexpressing *H19* mRNA, the cell migration was significantly increased (Figure 2A). On the contrary, in parental MDA-MB-231 and SUM159PT cells treated with siRNA targeting *H19*, the cell migration was significantly decreased (Figure 2B). In MDA-MB-231 cells stably overexpressing miR-675, the cell migration was significantly increased (Figure 2C). Similarly, parental SUM159PT cells transfected with miR-675 anti-miR exhibited decreased migration (Figure 2D). 

Then, we checked the migration abilities in the SUM159PT pH19-Cherry model. We observed a three-fold increase of the migration with the mCherry^high^ fraction compared to the mCherry^neg^ fraction (Figure 2E). As expected, the use of a miR-675 inhibitor reduced 25% of the migration in the mCherry^high^ fraction compared to the mCherry^neg^ control cells (Figure 2F). However, when we compared the cell migration with the miR-675 inhibitor in the mCherry^neg^ and mCherry^high^ fractions, we observed an increase of the migration in the presence of the miR-675 inhibitor in mCherry^high^ (Figure 2G). This suggests that, with a two-fold gene expression, the lncRNA *H19* per se is able to increase the migration independently of miR-675. 

**Figure 3:**
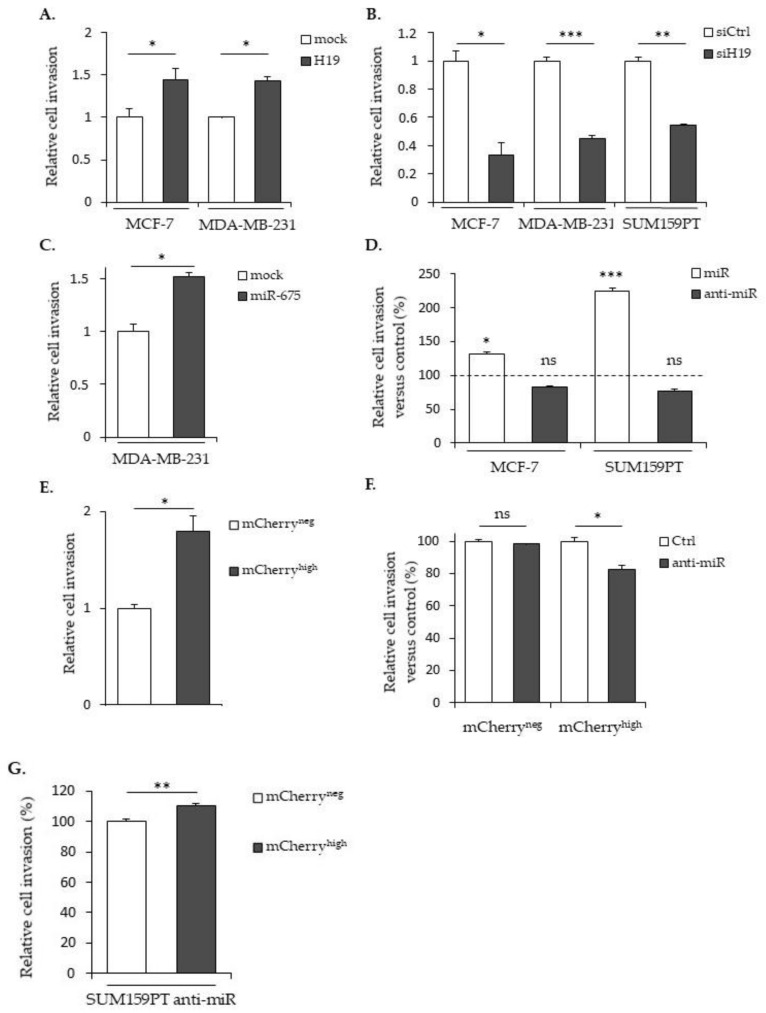
*H19* and miR-675 participate in the breast cancer cells invasion. (**A**) Control (mock) or *H19*-stably overexpressing cells (H19) were cultured in Transwell for 24 h. Invasive cells were then incubated with 1-mM Hoescht 33258 and counted. (**B**) Invasive capacities of the control (siCtrl) and *H19*-knockdowned cells (siH19) determined by Transwell assay. (**C)** MDA-MB-231 control (mock) or miR-675-stably overexpressing cells (miR-675) were cultured in Transwell for 24 h. Invasive cells were then incubated with 1-mM Hoescht 33258 and counted. (**D**) Invasive capacities of miR-675-transfected cells (miR) or miR-675-specific inhibitor transfected cells (anti-miR) determined by Transwell assay. Results are presented as the percentage of the control. (**E**) Relative invasive capacities of SUM159PT-pH19-mCherry^neg^ versus SUM159PT-pH19-mCherry^high^ determined by Transwell assay. (**F**) Invasive capacities of miR-675-specific inhibitor transfected cells (anti-miR). Results are presented as the percentage of the control. (**G**) Invasive capacities of miR-675-specific inhibitor transfected cells (anti-miR). Results are presented as a percentage of the mCherry^neg^ condition. * *p* < 0.05; ** *p* < 0.01; *** *p* < 0.001; ns: not significant.

We next determined the role of *H19* and miR-675 in the cell invasion by using Transwell previously coated with collagen. In MCF-7 or MDA-MB-231 cells stably overexpressing *H19* mRNA, the cell invasion was significantly increased compared to the mock cells (Figure 3A). Accordingly, *H19* knockdown using siRNA reduced the invasive capacities of parental MCF-7, MDA-MB-231 or SUM159PT cells (Figure 3B). The miR-675 expression significantly increased the invasion, as observed in MDA-MB-231 cells stably overexpressing miR-675 (Figure 3C) or in MCF-7 and SUM159PT cells transfected with miR-675 mimics (Figure 3D). The invasion of mCherry^high^ cells was also increased when compared to mCherry^neg^ cells (Figure 3E). The miR-675 inhibitor diminished the invasion of mCherry^high^ cells compared to the control cells (Figure 3F), highlighting the involvement of miR-675 in the *H19*-induced invasion. However, in the same way as for the cell migration, we observed an increase of the invasion in the presence of the miR-675 inhibitor in mCherry^high^ (Figure 3G), suggesting that the lncRNA *H19*-induced invasion does not require miR-675 action.

All together, these data show that both *H19* and miR-675 are able to promote the migration and invasion of breast cancer cells.

### 2.3. LncRNA H19 and miR-675 Differentially Regulate the Expression of Epithelial and Mesenchymal Markers 

**Figure 4:**
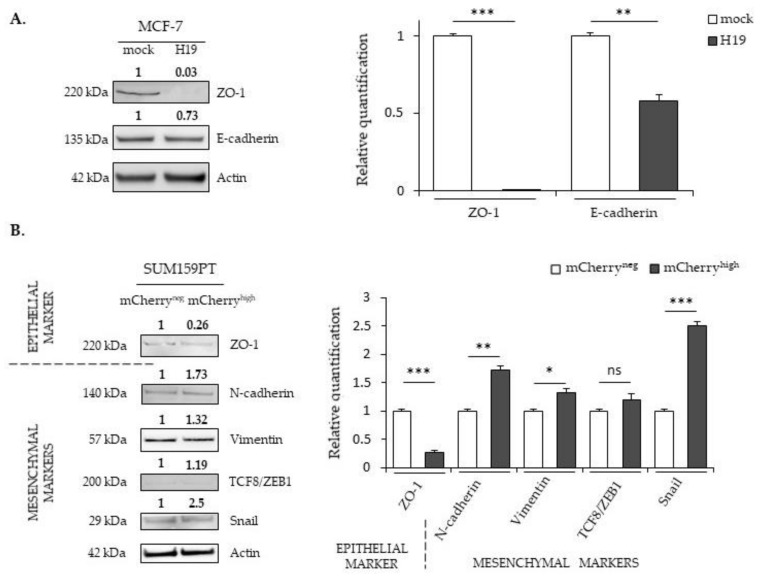

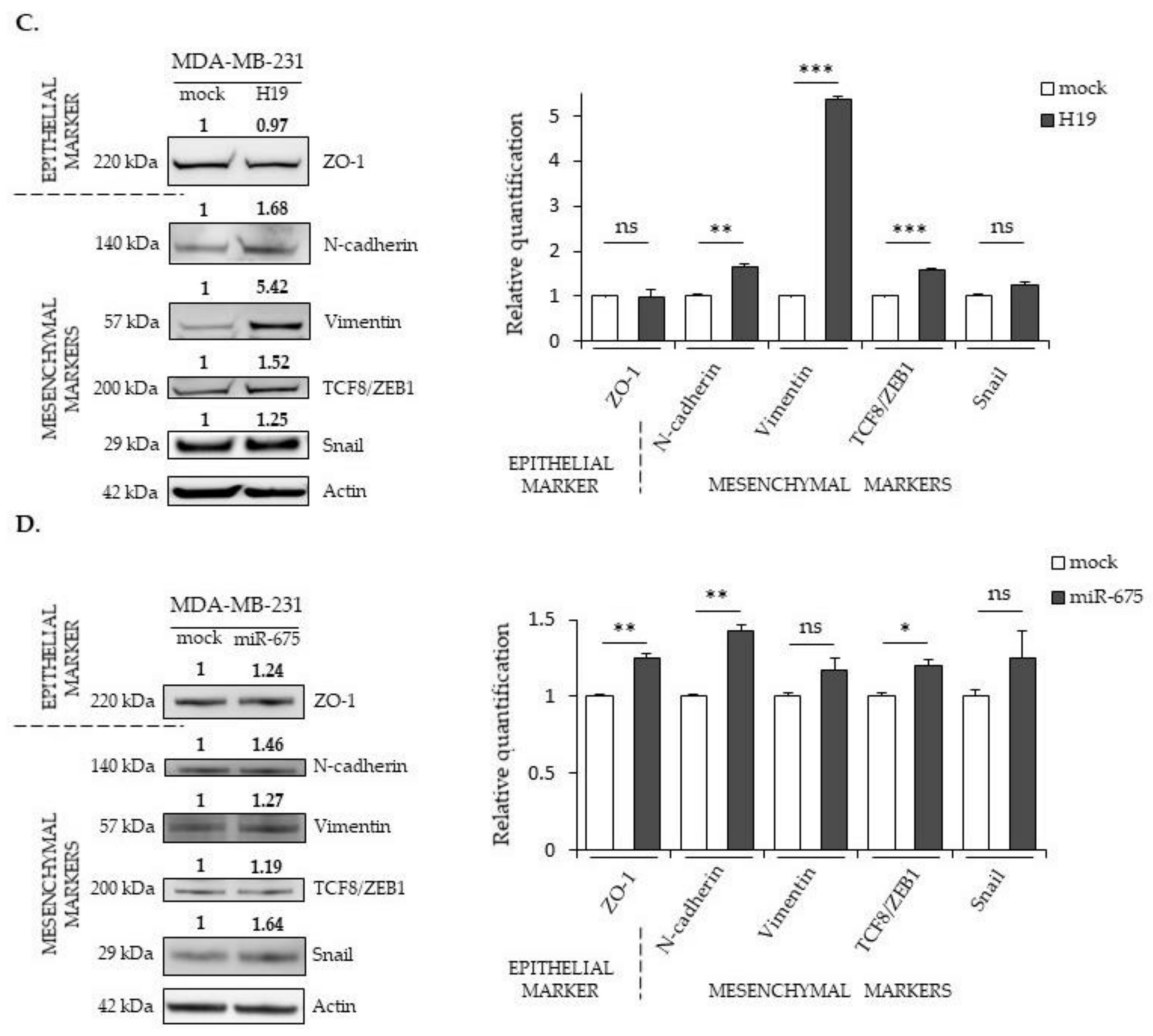
The effects of *H19* and miR-675 on the expressions of the epithelial-to-mesenchymal transition (EMT) markers. (A) EMT protein expressions in the MCF-7 control (mock) or *H19*-stably overexpressing cells (H19), determined by Western blot analysis. (B) EMT protein expressions in SUM159PT-pH19-mCherry^neg^ (mCherry^neg^) or SUM159PT-pH19-mCherry^high^ (mCherry^high^), determined by Western blot analysis. (C) EMT protein expressions in the MDA-MB-231 control (mock) or *H19*-stably overexpressing cells (H19), determined by Western blot analysis. (D) EMT protein expressions in the MDA-MB-231 control (mock) or miR-675-stably overexpressing cells (miR-675), determined by Western blot analysis. For each panel, actin was used as the equi-loading control. The relative signal intensities were quantified by ImageJ and shown above the protein bands for the representative experiment figure. The quantification of the triplicate is figured in the graph beside. * *p* < 0.05; ** *p* < 0.01; *** *p* < 0.001; ns: not significant. Uncropped blots are shown in Figures S1–S22.

Enhanced mobility and invasive abilities are required for cancer cells to invade the surrounding tissues and promote metastatic development. These properties are also part of the phenotype changes due to the epithelial-to-mesenchymal transition (EMT). The EMT is a process in which cells lose their epithelial characteristics to acquire new properties of mesenchymal cells. During the EMT, epithelial cells undergo dramatic molecular modifications with the downregulation of tight- and adherent-junction proteins such as ZO-1 and E-cadherin and the upregulation of specific markers of mesenchymal cells, including N-cadherin, vimentin and fibronectin. The expression of these markers is regulated by numerous transcriptional factors such as TCF8/ZEB1 and Snail. In a first approach to study the EMT process, we measured, by Western blot, the expression of molecular markers of epithelial or mesenchymal phenotypes (Figure 4). 

ZO-1 is a protein that forms tight junctions. It binds transmembrane proteins to the actin cytoskeleton. E-cadherin participates in intramembrane junctions to modify the cell morphology by modulating the cytoskeleton. The TCF8/ZEB1 transcription factor inhibits adhesion junctions by suppressing the expression of E-cadherin, whereas the Snail transcription factor suppresses ZO-1 expression. Vimentin is of mesenchymal origin; it modulates the structural dynamics and reorganizes the intermediate filaments. In MCF-7 cells displaying an epithelial phenotype, the expression of mesenchymal markers is not detected in whatever *H19* level. However, ZO-1 and E-cadherin proteins (epithelial markers) are less expressed in MCF-7 cells overexpressing *H19* compared to control cells (Figure 4A). In SUM159PT cells presenting less epithelial phenotypes than MCF-7 cells, a decrease of the ZO-1 expression and an increase of N-cadherin, vimentin, and Snail protein levels were observed in the mCherry^high^ cells compared to the mCherry^neg^ cells (Figure 4B). Interestingly, the expressions of mesenchymal markers (TCF8/ZEB1, N-cadherin, vimentin and Snail) were further enhanced in MDA-MB-231 cells stably overexpressing *H19* (Figure 4C). Together, these data indicate that, whatever the initial phenotypes of the cells, high levels of *H19* promote the EMT by decreasing the expression of epithelial markers and/or increasing the expression of mesenchymal markers.

The expression profile of the EMT markers in MDA-MB-231 cells stably overexpressing miR-675 was quite surprising, with an increased expression for both the epithelial marker ZO-1 and the mesenchymal markers TCF8/ZEB1 and N-cadherin (Figure 4D). In addition, vimentin and Snail expressions were not significantly modified (Figure 4D). These modifications suggest a hybrid phenotype induced by miR-675.

### 2.4. LncRNA H19 and miR-675 Promote Breast Cancer Colony Formation

**Figure 5:**
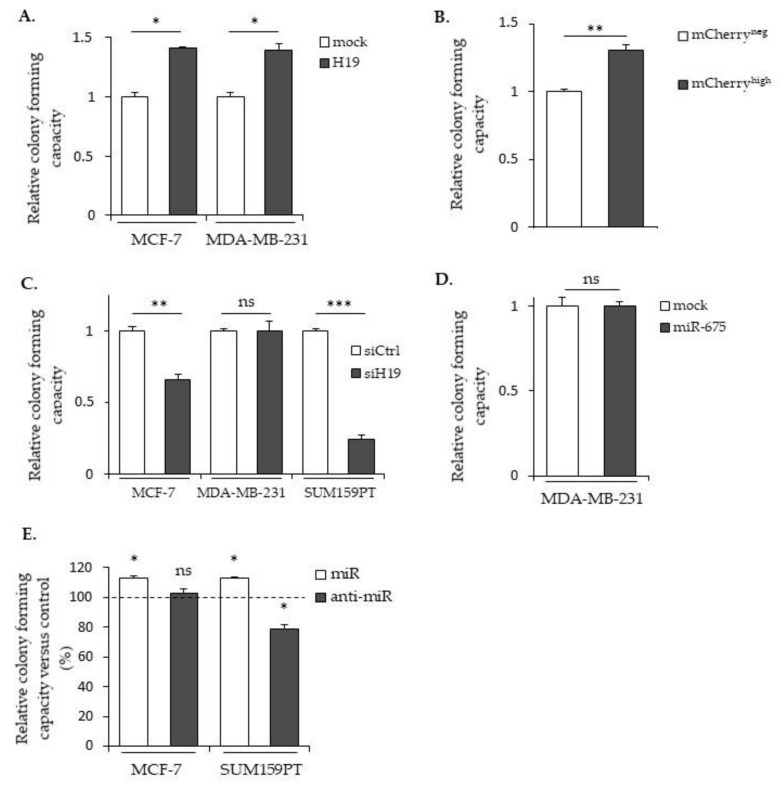
Effects of *H19* and miR-675 in colony formations. (**A**) The clonogenic capacities of the control (mock) or *H19*-overexpressing cells (H19). (**B**) The relative clonogenic capacities of SUM159PT-pH19-mCherry^neg^ versus SUM159PT-pH19-mCherry^high^. (**C**) The clonogenic capacities of the control (siCtrl) and *H19*-knockdown cells (siH19). (**D**) The clonogenic capacities of the MDA-MB-231 control (mock) or miR-675-overexpressing cells (miR-675). (**E**) The clonogenic capacities of the miR-675-transfected cells (miR) or miR-675-specific inhibitor transfected cells (anti-miR). Results are presented as a percentage of the control. * *p* < 0.05; ** *p* < 0.01; *** *p* < 0.001; ns: not significant.

The clonogenic assay is an in vitro cell survival assay based on the ability of a single cell to survive and grow into a colony, which is indicative of cancer cell aggressiveness. We thus performed clonogenicity assays with cells expressing different levels of *H19* and miR-675 (Figure 5). As shown in Figure 5A, MCF-7 and MDA-MB-231 cells stably overexpressing *H19* formed more colonies compared to the controls. Similar results were observed in mCherry^high^ compared to mCherry^neg^ SUM159PT cells (Figure 5B). Furthermore, *H19* knockdowns using siRNA induced a decrease in colony formation of native MCF-7 and of SUM159PT cell lines (Figure 5C). These results indicate that *H19* is able to enhance the clonogenicity of breast cancer cells. The effect of miR-675 in colony formations varied according to the cell lines used: MDA-MB-231 cells stably overexpressing miR-675 displayed a similar colony formation ability when compared to the control cells (Figure 5D). In contrast, miR-675 mimics increased colony formations in MCF-7 and SUM159PT cells, while anti-miR-675 decreased colony formations only in SUM159PT cells (Figure 5E). 

### 2.5. LncRNA H19 and miR-675 Enhance the Stemness Properties of Breast Cancer Cells 

**Figure 6:**
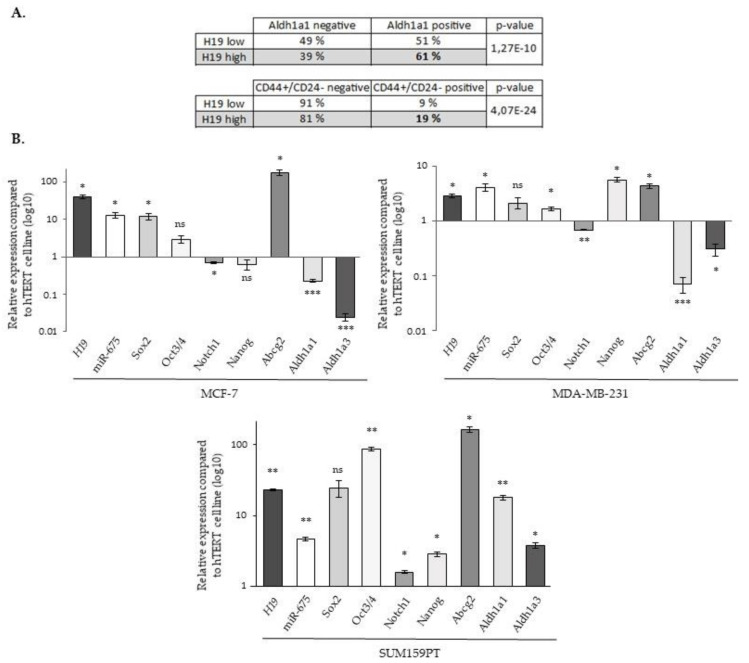
The expression of *H19*, miR-675 and different stem cell markers. (**A**) The expression of the *H19* gene in mammary tumors expressing or not stem cell signatures. The table represents the *H19* gene expression dependent on the gene signature of the tumors. Two gene signatures were used, one obtained in cell expressing ALDHA1 and the other in CD44_+_/CD24^-^. (**B**) The relative expression of *H19*, *Sox2*, *Oct3/4*, *Notch1*, *Nanog*, *Abcg2*, *Aldh1a1* and *Aldh1a3* genes and miR-675 in MCF-7, MDA-MB-231 and SUM159PT cells. The expression levels were related to the expression levels in hTERT cells indexed to 1. For panel A, a Fisher’s exact test was performed. * *p* < 0.05; ** *p* < 0.01; *** *p* < 0.001; ns: not significant.

To further investigate the involvement of lncRNA *H19* and miR-675 in breast cancer cell aggressiveness, we then investigated if lncRNA *H19* and miR-675 could endow the cells with stemness properties. For this, a meta-analysis on the gene expression data of more than 5000 breast tumor samples [25] was first carried out to explore the correlation of *H19* expression and that of the stem cell gene signature. Two published gene signatures were used, one obtained with cells expressing the ALDH1a1 isoform [26] and the other with cells harboring a combination of the overexpression of CD44 and a low expression of CD24 (CD44+/CD24-) [27]. Tumors were classified as *H19^high^* and *H19^low^* according to the expression of *H19* higher or lower, respectively, to the median expression value. For each stem cell signature tested, we observed a higher expression of *H19* in tumors expressing the signature, suggesting that lncRNA *H19* may play a role in breast cancer stem cells (Figure 6A).

We then analyzed by RT-qPCR the expression of the different markers involved in the cancer stem cells’ biology (*Sox2*, *Oct3/4*, *Notch1*, *Nanog*, *Abcg2*, *Aldh1a1* and *Aldh1a3*). As expected, a correlation was found between *H19* and miR-675. Of note, although the expressions of *H19* and miR-675 were generally correlated with that of cancer stem cell markers, some differences existed depending on the considered cell line. For example, diminutions of *Notch1*, *Aldh1a1* and *Aldh1a3* expressions were observed in the MCF-7 and MDA-MB-231 cell lines, contrary to SUM159PT cells. Similarly, the *Nanog* expression was increased in MDA-MB-231 and in SUM159PT cells but not in MCF-7 cells (Figure 6B). 

**Figure 7:**
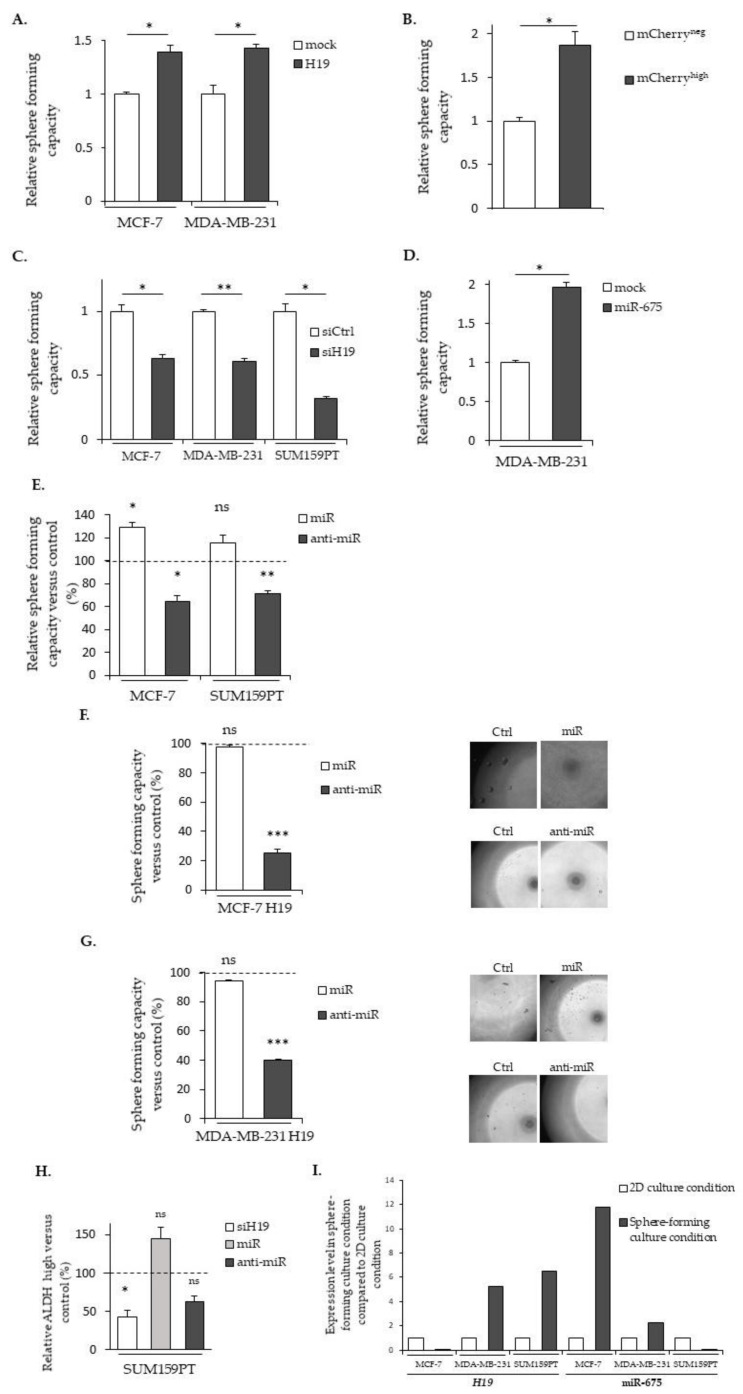
*H19* and miR-675 enhance the sphere formation of breast cancer cells. (**A**) Sphere-forming capacities of the control (mock) or *H19*-stably overexpressing cells (H19). (**B**) The relative sphere-forming capacities of SUM159PT-pH19-mCherry^neg^ versus SUM159PT-pH19-mCherry^high^. (**C**) The sphere-forming capacities of the control (siCtrl) and *H19*-knockdown cells (siH19). (**D**) The sphere-forming capacities of the MDA-MB-231 control (mock) or miR-675-stably overexpressing cells (miR-675). (**E**) The sphere-forming capacities of miR-675-transfected cells (miR) or miR-675-specific inhibitor transfected cells (anti-miR). Results are presented as a percentage of the control. (**F**) The sphere-forming capacities of MCF-7 *H19*-stably overexpressing cells transfected with miR-675 (miR) or the miR-675-specific inhibitor (anti-miR). Results are presented as a percentage of the control. Representative pictures for each condition are shown. (**G**) The sphere-forming capacities of MDA-MB-231 *H19*-stably overexpressing cells transfected with miR-675 (miR) or the miR-675-specific inhibitor (anti-miR). Results are presented as a percentage of the control. Representative pictures of each condition are shown. (**H**) ALDEFLUOR-positive subpopulations defined by the ALDEFLUOR assay in *H19*-knockdown cells (siH19), miR-675-transfected cells (miR) or miR-675-knockdown cells (anti-miR). Results are presented as a percentage of the ALDEFLUOR-positive subpopulation in the native cells. (**I**) The expression levels of *H19* and miR-675 in the cell lines cultured in sphere-forming conditions versus the same cell lines cultured in 2D conditions. * *p* < 0.05; ** *p* < 0.01; *** *p* < 0.001; ns: not significant.

The sphere-forming capacity of cells is used as the gold standard in an in vitro functional assay to analyze the ability of cancer stem cells to proliferate under anchorage-independent conditions in a defined medium [28]. As shown in Figure 7A, the MCF-7 and MDA-MB-231 cells stably overexpressing *H19* formed more spheres compared to the controls. Similarly, the ability of cells to form spheres was also increased in mCherry^high^ SUM159PT cells compared to mCherry^neg^ ones (Figure 7B). A decrease of the sphere-forming capacity was observed in the native three cell lines when *H19* was knocked down with siRNA (Figure 7C), confirming that *H19* was able to enhance the sphere-forming capacity of breast cancer cells. On the other hand, MDA-MB-231 cells stably overexpressing miR-675 also formed more mammospheres (Figure 7D). Similarly, the miR-675 mimic increased and the miR-675 inhibitor decreased the sphere-forming capacity of native MCF-7 and SUM159PT cells (Figure 7E), indicating that miR-675 was also involved in the sphere-forming capacity of breast cancer cells. Interestingly, the miR-675 mimic did not further increase the sphere formation in MCF-7 and MDA-MB-231 cells overexpressing *H19*. However, the anti-miR transient expression induced a decrease of the sphere formation by 70% in MCF-7 cells (Figure 7F) and by 60% in MDA-MB-231 cells *H19* (Figure 7G). Complementarily, we performed an ALDEFLUOR assay on the intact and viable cells after the siRNA *H19*, miR-675 mimic and miR-675 inhibitor transient expressions. The siRNA *H19* induced a diminution of the ALDH^high^ subpopulation compared to the control cells, suggesting that *H19* enriched for breast cancer stem cells. Similar trends, though not significant, were observed in cells that were transfected with the miR-675 mimic or inhibitor (Figure 7H). 

Enhanced sphere formations by *H19* and miR-675 prompted us to determine their expressions in native cells under culture conditions favoring stem cell proliferations. We observed an increased expression of *H19* in MDA-MB-231 and SUM159PT and an increased expression of miR-675 in MCF-7 in these conditions when compared to a monolayer culture (Figure 7I). 

Collectively, these results demonstrate the involvement of *H19* and miR-675 in the enrichment of cancer stem cells. 

## 3. Discussion

The long non-coding RNA *H19* has been shown to intervene at multiple steps of tumorigenesis, such as cell proliferation, migration, invasion, induction of the epithelial-to-mesenchymal transition and metastasis [13]. However, several data indicate that *H19*-derived miR-675 could also be implicated in these oncogenic cellular processes [20,22,23]. Our work focuses on the roles of both *H19* and miR-675 in metastasis-related phenotypes such as migration, invasion, the EMT, colony formation and stemness properties.

We assessed the breast cancer invasion by the transplantation of cells in zebrafish embryos. Indeed, cell lines from a variety of human cancers, including metastatic melanoma, pancreatic, ovarian, breast, glioma and colorectal cell lines, are capable of proliferating, invading and forming tumor masses in the zebrafish embryo during xenotransplantation studies [29,30]. The Tg(fli1:GFP) transgenic line used in this study allowed us to visualize blood vessels in the living host and to allow for the analysis of cancers cells after their intravasation into the blood vessels. We demonstrated that both *H19* and miR-675 overexpressing cells were able to more efficiently colonize zebrafish. 

In vitro, *H19* and miR-675, alone or together, participate in cellular migration and invasion; migration and invasion are enhanced by both the miR-675 mimic transient expression and miR-675 overexpression but, also, by *H19* overexpression. On the contrary, decreases in the migration and invasion are observed upon *H19*-silencing or miR-675-inhibitor transient expressions. However, we observed that the miR-675 inhibitor has different effects according to the cell types, probably reflecting their basal levels of *H19* expressions or their molecular classifications. Indeed, the MCF-7 cell line is a luminal-like subtype, whereas the MDA-MB-231 cell line is a triple-negative subtype. To reinforce this hypothesis, it has been shown that *H19* is associated with a poor prognosis in triple-negative breast cancer patients [31]. 

**Figure 8:**
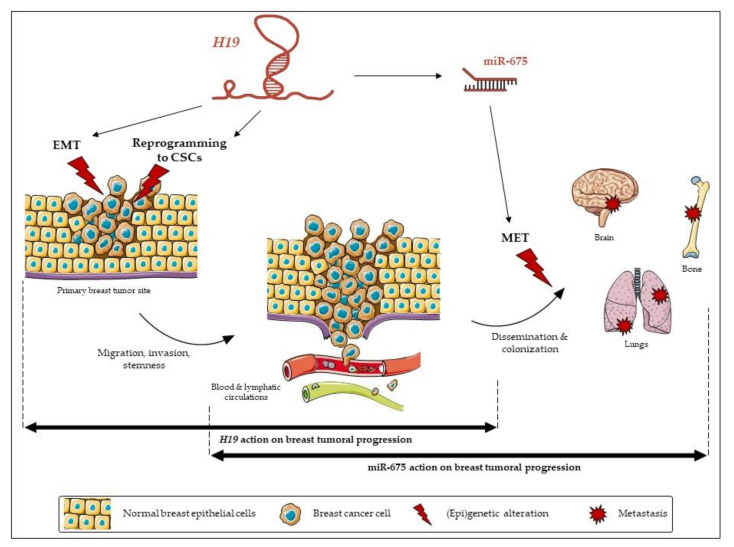
The relative contribution of long non-coding (lnc)RNA *H19* and its miR-675 in breast cancer progression.

Both *H19* and miR-675 are able to modulate the expressions of the EMT markers, although their actions differ according to the cell types. In the MCF-7 cell line, *H19*-overexpressing cells displayed decreased expressions of epithelial markers. By contrast, in MDA-MB-231 and in SUM159PT pH19-mCherry^high^ cells, high levels of *H19* were associated with increased expressions of mesenchymal markers, and, very interestingly, SUM159PT pH19-mCherry^high^ cells also displayed decreased expressions of the epithelial marker ZO-1. Our results are in agreement with the work of Liao et al., who demonstrated that the lncRNA *H19* induces the proliferation and invasion of lung cancer cells via the overexpression of N-cadherin and vimentin and the decrease of E-cadherin [32]. In MDA-MB-231 cells, miR-675 promotes ZO-1 and N-cadherin expressions. These results highlight an interesting difference of action between *H19* and miR-675. In the same cell line, *H19* favors the expression of EMT markers, whereas the action of miR-675 seems ambiguous by favoring the expression of both mesenchymal and epithelial markers, suggesting the role of miR-675 in both the EMT and mesenchymal-to-epithelial transition (MET). Indeed, the miR-675 alone does not induce the same molecular determinants as *H19*. These data reveal that *H19* and miR-675 participate in both the migration and invasion, yet *H19* and miR-675 do not exert the same role during the molecular events of the EMT. The MET is essential to colonize and proliferate in the different stages of metastasis [33]. Thus, *H19* and miR-675 could have sequential effects leading to metastasis; *H19* would favor the departure of cells from the primary tumor by promoting the EMT, while miR-675 would also favor a metastatic colonization and the development of secondary tumors by inducing the MET (Figure 8). Alternately, an intermediate status between the epithelial and mesenchymal phenotypes, i.e., the hybrid epithelial-mesenchymal state, is increasingly described to be involved in migration and invasion. The hybrid phenotype endows cancer cells with a more plastic status to adapt the stressful environment for the metastasis formation [34]. Indeed, the hybrid phenotype may contribute to the cancer collective cell migration and, in fine, to metastasis [35]. Our data suggested that miR-675 could favor the acquisition of this hybrid phenotype to favor metastases.

The epithelial-to-mesenchymal transition is an important cellular mechanism corresponding to a differentiation from an epithelial cell to a mesenchymal-like cell. It involves multiple molecular pathways [36]. The EMT appears essential for embryonic development and tissue repair but, also, for the progression of cancer and metastases [37]. The role of miR-675 in the EMT is not described, and the role of *H19* in the EMT appears contradictory. Numerous data indicate that *H19* promotes the EMT [38,39,40] in various tissues, including the breast [41], but Zhang and collaborators demonstrated that *H19* is implied in the metastasis suppression of hepatocellular carcinoma [42]. Our results showing the contrary effects of *H19* and miR-675 could explain these discrepancies.

As previously indicated, the lncRNA *H19* can act at multiple levels of regulation, more particularly by sponging microRNAs. For instance, Lv et al. demonstrated that the upregulation of *H19* promotes the migration and invasion in bladder cancer by sponging miR-29b-3p. However, the knockdown of *H19* allowed miR-29b-3p to facilitate the MET [43]. It is also known that the miR-200 family (miR-200a, miR200b, miR-200c, miR-141 and miR-429) is involved in the EMT via the ZEB1/E-cadherin pathway [44,45]. Indeed, these microRNAs regulate ZEB1 and ZEB2 proteins through the repressed E-cadherin expression. ZEB1 and ZEB2 are transcriptional factors implicated in metastasis. When the miR-200 family is inhibited, the EMT is induced, and, conversely, the expression of these microRNAs leads to the MET [46]. Many studies show that *H19* inhibits members of miR-200 family [47]. Indeed, *H19* sponges miR-200a or miR-200b/c to promote cancer metastasis through ZEB1 and ZEB2 upregulations [48,49]. 

Complementarily, we explored the role of *H19* and miR-675 in the resistance to anoikis. Anoikis is a cell death induced by the detachment of a cell from the extracellular matrix. This cellular phenotype is involved in the metastatic development; indeed, during invasion, cells have to break their adhesions so that they can reach the secondary tumor site. To evaluate the role of *H19* and miR-675 in this process, we studied the resistance to anoikis in breast cancer cell lines overexpressing *H19* or miR-675. The anoikis resistance assay in MCF-7 cells stably overexpressing or not *H19* does not show any long-term statistical differences. In the same way, the viability of MDA-MB-231 cells overexpressing *H19* or miR-675 does not show any significant variation compared to the control.

In connection with the phenotypes linked to the EMT, we further investigated the involvement of *H19* in the regulation of breast cancer stem cells. Indeed, *H19* has already been associated with stemness in breast cancer [16,31,50], in part by favoring symmetric division. In this report, we highlight a correlation between the expression of the *H19* gene and the presence of stem cell markers in a cohort of more than 5000 breast cancer clinical samples. A gene signature of cells expressing two stemness markers, CD44+/CD24− and ALDH1A1 [51,52], is correlated with the *H19* expression. In addition, our results indicate that the overexpression of *H19* and miR-675 observed in cancer cells is accompanied overall with an overexpression of different stem cell markers such as *Sox2*, *Oct3/4* and *Abcg2* in breast cancer cell lines. We observed an increase of *Nanog* only in MDA-MB-231 and SUM159PT triple-negative breast cancer cells and an increase of *Notch1*, *Aldh1a1* and *Aldh1a3* only in the SUM159PT cell line. In these latter cells, the activity of ALDH is decreased with siRNA-targeting *H19* and with the miR-675 inhibitor but is enhanced by the transient expression of the miR-675 mimic. To our knowledge, it is the first time that the role of miR-675 on cancer cells’ stemness has been described. *Nanog*, *Sox2* and *Oct3/4* are transcription factors largely described in the literature for their involvement in the maintenance of self-renewal and pluripotency of embryonic stem cells [53]. Their implication in cancer stem cells’ regulation has also been demonstrated [54]. *Abcg2* is an efflux pump associated with the drug resistance of cancer stem cells [54]. A transcriptional regulatory network involving *Sox2*, *Oct3/4* and c-Myc ensures the maintenance of the stem cell pluripotency [53]. Interestingly, c-Myc has been shown to bind alleles specifically upstream of the promoter of the *H19* gene in order to promote its transcription [55]. Similarly, *Sox2* and *Oct3/4* are able to bind upstream of the *H19* gene [56]. This fixation prevents the promoter methylation on the maternal allele and, therefore, contributes to the gene expression. The control of the *H19* gene by these three factors suggests that *H19* may be a factor involved in the stem cell regulation.

It is often described that miRNAs have a similar role to the host genes that produce it. However, in several cases, miRNAs have antagonistic actions against its host gene [57]. For instance, in breast cancer, miR-301 derived from *SKA2* gene, which has an oncogenic action, negatively regulates tumor suppressors such as PTEN [58]. Conversely, miR-483-5p, derived from the *IGF2* gene, inhibits angiogenesis, unlike its host gene [59]. In our work, we show a similar role and/or antagonist actions of miR-675 and its host gene *H19,* depending on the phenotype studied. Indeed, *H19* and miR-675 have similar functions during the cellular migration and invasion and seem to regulate stemness in the same manner. By contrast, their role would be antagonistic during the epithelial-to-mesenchymal transition and colony formation. 

We show that miR-675 modulates the phenotypes associated with the occurrence of metastases in breast cancer cells. However, miR-675 generates two mature miRNAs (miR-675-5p and miR-675-3p), both of which have different targets [60]. A logical continuation of this work will therefore be to determine which mature miRNA is associated with these phenotypes. 

In continuation of this investigation, it would be interesting to study the molecular targets involved in the occurrence and the development of breast cancer metastases. Despite the similarity of the phenotypes observed, lncRNA *H19* and miR-675 may not have the same molecular determinants and, therefore, use distinct signaling pathways. LncRNA *H19* induces the cell migration and invasion by different mechanisms, depending on the cancers. Indeed, *H19* promotes the migration and invasion of colon cancer cells via the MAPK signaling pathway [61] and those of human osteosarcoma through the NF-κB pathway [62]. It would be relevant to investigate if these signaling pathways are similarly activated in breast cancer cells, as a better knowledge of the molecular determinants associated with the *H19* and miR-675 phenotypes in breast cancer progression would provide new therapeutic opportunities.

## 4. Materials and Methods 

### 4.1. Cell Culture

The breast cancer cell lines MCF-7 estrogen-sensitive, MDA-MB-231 and SUM159PT estrogen-insensitive were maintained routinely in Dulbecco’s modified Eagle’s medium (DMEM, Gibco), Roswell Park Memorial Institute medium (RPMI, Gibco) and Ham’s F-12 Nutrient Mix (Gibco), respectively, containing 10% fetal bovine serum (FBS) and 0.01% ZellShield (Clinisciences). F-12 medium was supplemented with 500 µL of insulin (10 mg/mL), 27.8 µL of hydrocortisone (100 mg/mL and 5 mL of HEPES 1M. Each cell lines were obtained from the American Type Culture Collection and cultured at 37 °C with 5% CO_2_ and 95% of air-humidified atmosphere. The MCF-7 and MDA-MB-231 cells arose from a pleural effusion of patients with metastatic breast adenocarcinoma. The SUM159PT cells came from a pleural effusion of patients with pleomorphic breast carcinoma. The immortalized cell line, hTERT, was cultured in MEGM (Lonza, Levallois, France) supplemented with 1% penicillin/streptomycin and served as the controls for the relative expression of the pluripotency genes. 

### 4.2. Establishment of H19 and miR-675 Overexpressing Cell Lines

To establish the cell lines overexpressing *H19*, the SUM159PT cell line was transfected with 1 µg of pH19-mCherry using Nucleofector Amaxa (Lonza), and cells were allowed to recover for 48 h. Cells were then selected in the presence of hygromycin B at 0.6 mg/mL for at least one month before their use for in vitro experiments. The *H19* overexpressing MCF-7 and MDA-MB-231 cell lines and the miR-675 overexpressing MDA-MB-231 cell line were previously described [23].

### 4.3. siRNA and miRNA Transfection

For siRNA transfection, 1 × 10^5^ cells were plated in 6-well plates. After 24h, cells were transfected with negative control (SR-CL000-005; Eurogentec) or *H19* siRNA (Table S1) using jetPRIME^®^ Transfection Reagent, according to the manufacturer’s guidelines (Polyplus Transfection^®^). Cells were lysed 48 h after transfection.

For miRNA transfection, 1 × 10^5^ cells were plated in 6-well plates. After 24 h, cells were transfected with Hsa-miR-675-5p mimic or its hairpin inhibitor with DharmaFECT-Duo, according to the manufacturer’s guidelines (Thermo Fischer Scientific Dharmacon). Cells were lysed 48 h after transfection.

### 4.4. RNA Extraction, Reverse Transcription and Real-time RT-PCR

RNA extraction and qRT-PCR were performed as previously described [63]. Primers used for qRT-PCR are described in Table S2.

miRNA extraction and qRT-PCR were performed as previously described [23].

### 4.5. Migration and Invasion Assays

Cell migration and invasion were determined by Transwell assay. For migration assay, 1 × 10^4^ cells were seeded on a 0.045-mg/mL collagen (Millipore)-coated insert (0.8 µM; BD Biosciences) of 12-well plates. After 8 h, cells migrating to the other side of the filter were stained with Hoescht 33258 1 mM and counted. For the invasion assay, 4 × 10^4^ cells were seeded on a 3-mg/mL collagen (Millipore)-coated insert (0.8 µM; BD Biosciences) of 12-well plates. After 24 h, cells invading to the other side of the filter were stained with Hoescht 33258 1 mM and counted.

### 4.6. Colony-forming and Sphere-forming Capacities

In the clonogenic assay, 250 cells were seeded in 100-mm² dishes. After 15 days, wells were fixed with 4% paraformaldehyde and stained with 0.5% crystal violet and counted.

For the sphere-forming capacity, a ranging from 1024 cells to 1 cell were cultured in a sphere medium consisting of phenol red-free DMEM-F12 (Gibco), 0.4% bovine serum albumin (Sigma-Aldrich, St Quentin Fallavier, France), 10 mL of B27 additive (Invitrogen, Illkirch, France), 5 mg/mL of insulin (Sigma-Aldrich), 4 µg/mL of heparin and 20 ng/mL of epidermal growth factor (EGF) and fibroblast growth factor (FGF) (Biotechne, Abingdon, OX, United Kingdom). Cells were seeded in 96-well low-adhesion plates. The number of spheres per well were counted 4 days later.

### 4.7. Aldefluor Assay

To measure the ALDH1 enzyme activity, 1 × 10^6^ cells were suspended in a ALDEFLUOR assay buffer containing ALDH1 substrate (BAAA, 1 µmol/L; StemCell) and incubated for 30 min at 37 °C. In each experiment, a sample of cells was incubated, under identical conditions, with 50 mmol/L of diethylaminobenzaldehyde as a negative control. Only the 30% most-negative cells were collected as ADLH- cells. Flow cytometry data were acquired on a CyAn ADP cytometer (Beckman Coulter, Villepinte, France) with Summit software. All analyses were performed with FlowJo software.

### 4.8. Western Blot Analysis

Cell lysis, electrophoresis, protein transfer, immunoblotting and signal revelation were performed as previously described [23]. Primary antibodies used were anti-E-cadherin, anti-N-cadherin, anti-Snail, anti-TCF8/ZEB1, anti-vimentin, anti-ZO-1 (#9782; Cell Signaling) and anti-actin (A2066-2ML; Sigma, St Quentin Fallavier, France) as a loading control.

### 4.9. Transgenic Zebrafish Xenograft 

SUM159PT pH19-mCherry^neg/high^ and MDA-MB-231 cells were incubated for 40 min in an atmosphere at 37 °C and 5% CO_2_, with a solution of lipophilic tracers (Vybrant DiI labeling solution for mCherry^high^ and for MDA-MB-231 cells overexpressing *H19* and miR-675, Vybrant DiD labeling solution for mCherry^neg^ and, for each, a MDA-MB-231 control; Invitrogen) prepared in 1 mL of culture medium without FBS. Then, cells were dissociated with trypsin/0.25% EDTA (Gibco) and counted. One million cells of each condition were centrifuged at 100 *g* for 5 min. The pellet was resuspended with 100 µL of PBS/EDTA 5 mM. From the laying, transgenic zebrafish (fli1:GFP) were placed in 0.2-mM 1-phenyl-2-thio-urea (Sigma) to prevent pigmentation up to 48 h postfertilization. Fish were anaesthetized with 0.04-mg/mL MS-222 (Sigma, A5040), and SUM159PT pH19-mCherry^neg^ (DiD) and pH19-mCherry^high^ (DiI) cells or MDA-MB-231 control cells (DiD) and MDA-MB-231-overexpressed *H19* or MDA-MB-321 control cells (DiD) and MDA-MB-231-overexpressed miR-675 cells (DiI) were coinjected using a stereomicroscope (LEICA M125) and a microinjector (FemtoJET; Eppendorf) in a yolk sac. After injection, zebrafish were placed in a solution of phenylthiourea and penicillin-streptomycin renewed every 2 days at 28 °C in the dark. During 48 h, the incubation temperature of the zebrafish varied (30 °C at 24 h and 32 °C at 48 h up to 3 days postinjection) to promote cell development. Fish were fixed with 4% paraformaldehyde solution and placed in Petri dishes with glass bottoms. Fluorescent pictures were captured using automated image acquisition software from ZEISS with a LSM 880 microscope. Transgenic Tg(fli1:EGFP)^y1^ zebrafish [64] were maintained in compliance with the French and European Union guidelines for the handling of laboratory animals (Directive 2010/63/EU of the European Parliament and of the Council of 22 September 2010 on the protection of animals used for scientific purposes). The experimental procedures carried out on zebrafish were reviewed and approved by the local ethics committee, CEEA 75 Nord Pas-de-Calais and the French Ministry of Higher Education and Research (APAFiS approval number 13527-2018011722529804_v3).

### 4.10. Statistical Analysis

Data are expressed as the mean values ± standard error of the mean of at least 3 independent experiments. The statistical analysis was done by using a Student’s *t*-test, and a *p*-value < 0.05 was considered significant. * *p* < 0.05, ** *p* < 0.01 and *** *p* < 0.001.

## 5. Conclusions

In conclusion, this work highlights the involvement and relative contribution of lncRNA *H19* and miR-675 in the occurrence and development of breast cancer metastasis. In most cases, *H19* and miR-675 exert similar effects. Indeed, we show that both *H19* and miR-675 participate in the migration, invasion and stemness of breast cancer cells. Yet, miR-675 does not have the same effect as the lncRNA *H19* during the EMT. Their actions appear to be complementary and contribute to the metastasis development. These results highlight the interactions between lncRNA and miRNA to influence the EMT and, after further validation, might be taken into account in the therapy of breast cancer. 

## Supplementary Materials

The following are available online at https://www.mdpi.com/2072-6694/12/7/1730/s1, Figure S1: Uncropped western blot membranes (visible condition) corresponding to Figure S2, Figure S2: Revelation of uncropped western blot membranes showing the detection of ZO-1 protein expression in MCF-7 control (mock) or *H19*-stably overexpressing cells (H19), and in MDA-MB-231 control (mock), *H19*-stably overexpressing cells (H19) or miR-675-stably overexpressing cells (miR-675). Actin was used as an equiloading control, Figure S3: Uncropped western blot membranes (visible condition) corresponding to Figure S4, Figure S4: Revelation of uncropped western blot membranes showing the detection of E-cadherin protein expression in MCF-7 control (mock) or H19-stably overexpressing cells (H19). Actin was used as an equiloading control. Figure S5: Uncropped western blot membranes (visible condition) corresponding to Figure S6. Figure S6: Revelation of uncropped western blot membranes showing the detection of TCF8/ZEB1 protein expression in MDA-MB-231 control (mock), H19-stably overexpressing cells (H19) or miR-675-stably overexpressing cells (miR-675). Actin was used as an equiloading control, Figure S7: Uncropped western blot membranes (visible condition) corresponding to Figure S8, Figure S8: Revelation of uncropped western blot membranes showing the detection of N-cadherin protein expression in MDA-MB-231 control (mock), H19-stably overexpressing cells (H19) or miR-675-stably overexpressing cells (miR-675). Actin was used as an equiloading control, Figure S9. Uncropped western blot membranes (visible condition) corresponding to Figure S10, Figure S10: Revelation of uncropped western blot membranes showing the detection of vimentin protein expression in MDA-MB-231 control (mock), H19-stably overexpressing cells (H19) or miR-675-stably overexpressing cells (miR-675). Actin was used as an equiloading control, Figure S11: Uncropped western blot membranes (visible condition) corresponding to Figure S12, Figure S12: Revelation of uncropped western blot membranes showing the detection of snail protein expression in MDA-MB-231 control (mock), H19-stably overexpressing cells (H19) or miR-675-stably overexpressing cells (miR-675). Actin was used as an equiloading control, Figure S13: Uncropped western blot membranes (visible condition) corresponding to Figure S14, Figure S14: Revelation of uncropped western blot membranes showing the detection of ZO-1 protein expression in SUM159PT-pH19-mCherryneg (mCherryneg) or SUM159PT-pH19-mCherryhigh (mCherryhigh) cells. Actin was used as an equiloading control, Figure S15: Uncropped western blot membranes (visible condition) corresponding to Figure S16, Figure S16: Revelation of uncropped western blot membranes showing the detection of TCF8/ZEB1 protein expression in SUM159PT-pH19-mCherryneg (mCherryneg) or SUM159PT-pH19-mCherryhigh (mCherryhigh) cells. Actin was used as an equiloading control, Figure S17: Uncropped western blot membranes (visible condition) corresponding to Figure S18, Figure S18: Revelation of uncropped western blot membranes showing the detection of N-cadherin protein expression in SUM159PT-pH19-mCherryneg (mCherryneg) or SUM159PT-pH19-mCherryhigh (mCherryhigh) cells. Actin was used as an equiloading control, Figure S19: Uncropped western blot membranes (visible condition) corresponding to Figure S20, Figure S20: Revelation of uncropped western blot membranes showing the detection of vimentin protein expression in SUM159PT-pH19-mCherryneg (mCherryneg) or SUM159PT-pH19-mCherryhigh (mCherryhigh) cells. Actin was used as an equiloading control, Figure S21: Uncropped western blot membranes (visible condition) corresponding to Figure S22, Figure S22: Revelation of uncropped western blot membranes showing the detection of snail protein expression in SUM159PT-pH19-mCherryneg (mCherryneg) or SUM159PT-pH19-mCherryhigh (mCherryhigh) cells. Actin was used as an equiloading control, Table S1: References of H19 siRNA transient transfection, Table S2. Primer used for qRT-PCR.
