# Supplementary material for: Enhancement of Breast Cancer Cell Aggressiveness by lncRNA H19 and its Mir-675 Derivative: Insight into Shared and Different Actions
Source: Cancers (Basel). 2020 Jun 29;12(7):1730. doi: 10.3390/cancers12071730 (PMC7407157; doi:10.3390/cancers12071730)
Supplement: Supplementary file 1 [file cancers-12-01730-s001.pdf]

cancers

MDPI

Supplementary Materials: Enhancement of Breast Cancer Cell Aggressiveness by lncRNA H19 and its miR-675 Derivative: Insight into Shared and Different Actions

Evodie Peperstraete, Clément Lecerf, Jordan Collette, Constance Vennin, Ludivine Raby, Pamela Völkel, Pierre-Olivier Angrand, Marie Winter, François Bertucci, Pascal Finetti, Chann Lagadec, Samuel Meignan, Roland P. Bourette, Xuefen Le Bourhis and Eric Adriaenssens

Table S1. References of *H19* siRNA transient transfection.

siRNA	References
siNeg	SR-CL000-005 (Eurogentec)
siH19	ACGAGGCCAGGUCUCCAGCUG

Table S2. Primers used for qRT-PCR.

Primers	Sequence 5′ to 3′
RPLP0 reverse	GATGACCAGCCCAAAGGAGA
RPLP0 forward	GTGATGTGCAGCTGATCAAGACT
H19 reverse	TACAACCACTGCACTACCTG
H19 forward	TGGCCATGAAGATGGAGTCG
Sox2 reverse	CGGGGCCGGTATTTATAATC
Sox2 forward	TAACCCCAAGATGCACAACTC
Oct 3/4 reverse	GTGAAGTGAGGGCTCCCATA
Oct3/4 forward	GAAGGATGTGGTCCGAGTGT
Abcg2 reverse	GCTGCAAAGCCGTAAATCCATATC
Abcg2 forward	ACAACCATTGCATCTTGGCTGTC
Notch1 reverse	CTGTGAGGAAAATATCGAC
Notch1 forward	GTACTGACCTGTCCACTCT
Nanog reverse	ACACAGTGGGCCTGAAGA
Nanog forward	GTGATTTGTGGGCCTGAAGA
Aldh1a1 reverse	CCCACTCTCAATGAGGTCAAG
Aldh1a1 forward	GTTGAGCGGGCTAAGAAGTAT
Aldh1a3 reverse	ATAAAGGGCGGTGAGAGGTGTCT
Aldh1a3 forward	ACCATCCCCACAGATGACAAC

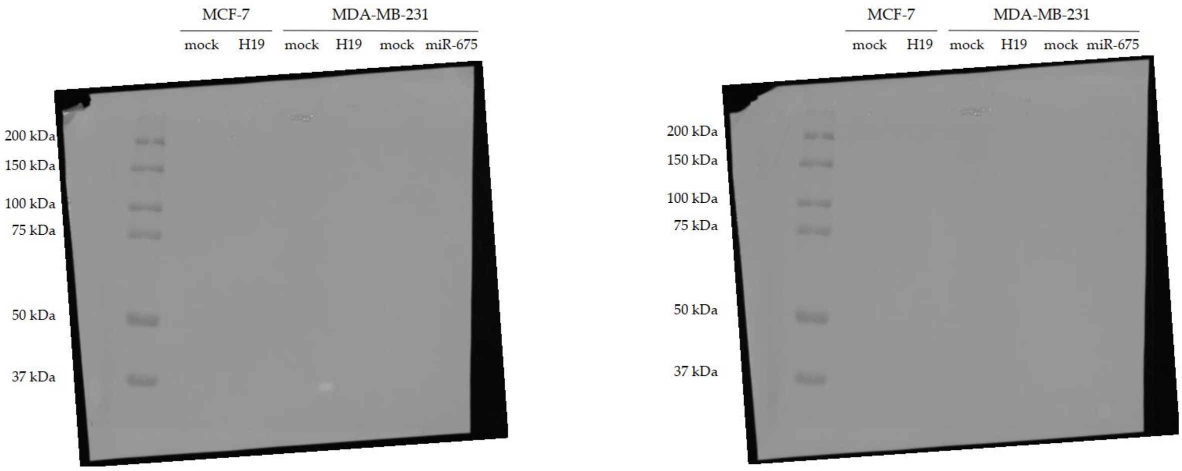

Figure S1. Uncropped western blot membranes (visible condition) corresponding to Figure S2.

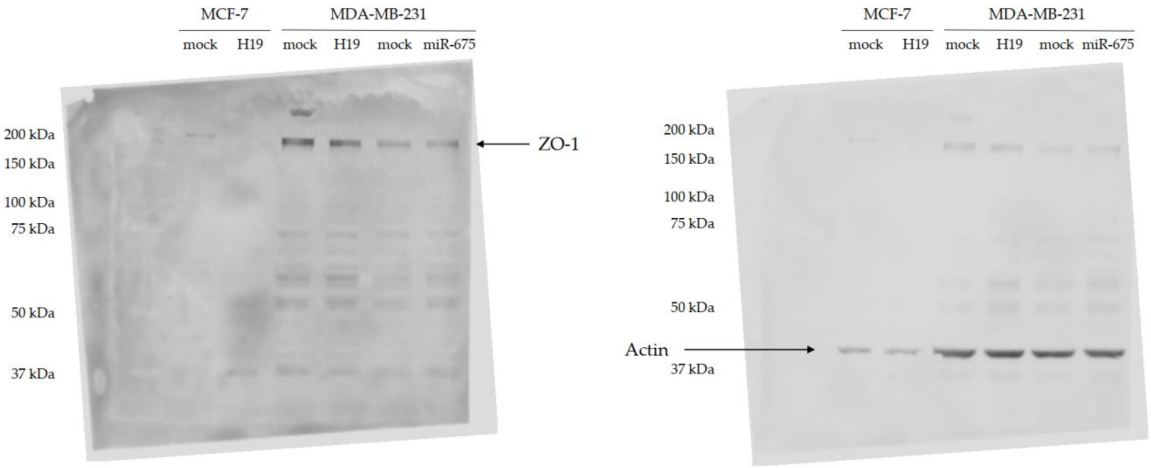

Figure S2. Revelation of uncropped western blot membranes showing the detection of ZO-1 protein expression in MCF-7 control (mock) or *H19*-stably overexpressing cells (H19), and in MDA-MB-231 control (mock), *H19*-stably overexpressing cells (H19) or miR-675-stably overexpressing cells (miR-675). Actin was used as an equiloading control.

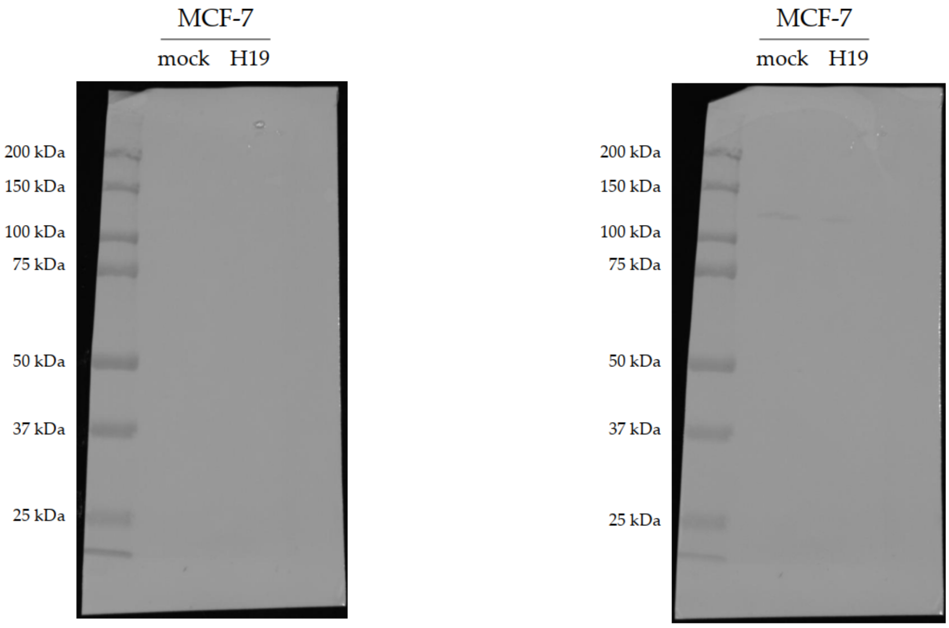

Figure S3. Uncropped western blot membranes (visible condition) corresponding to Figure S4.

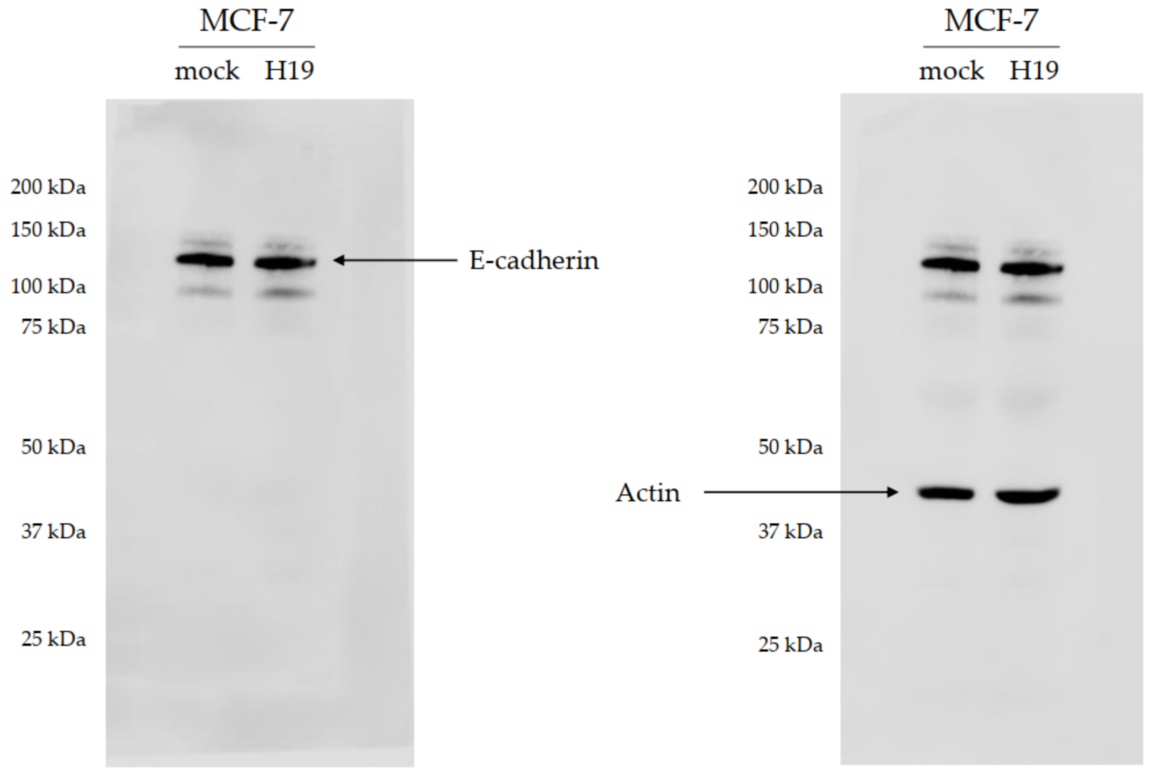

Figure S4. Revelation of uncropped western blot membranes showing the detection of E-cadherin protein expression in MCF-7 control (mock) or *H19*-stably overexpressing cells (H19). Actin was used as an equiloading control.

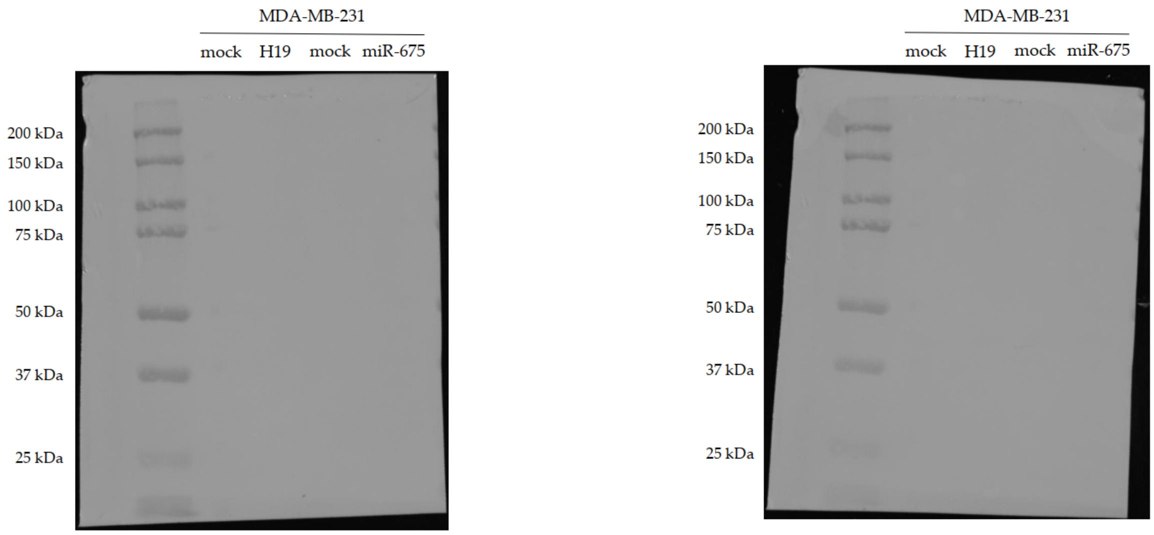

Figure S5. Uncropped western blot membranes (visible condition) corresponding to Figure S6.

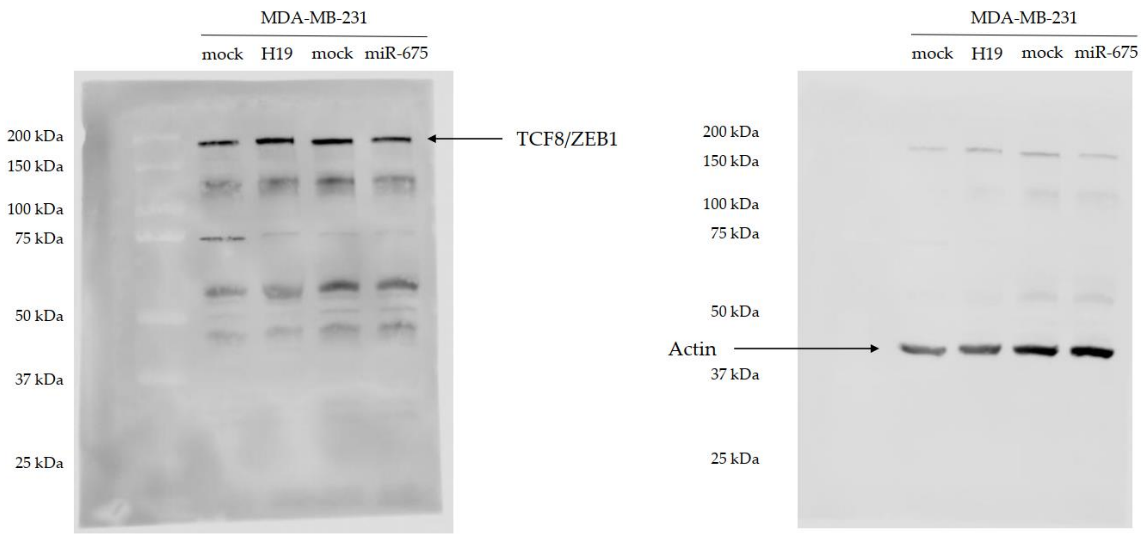

Figure S6. Revelation of uncropped western blot membranes showing the detection of TCF8/ZEB1 protein expression in MDA-MB-231 control (mock), *H19*-stably overexpressing cells (H19) or miR-675-stably overexpressing cells (miR-675). Actin was used as an equiloading control.

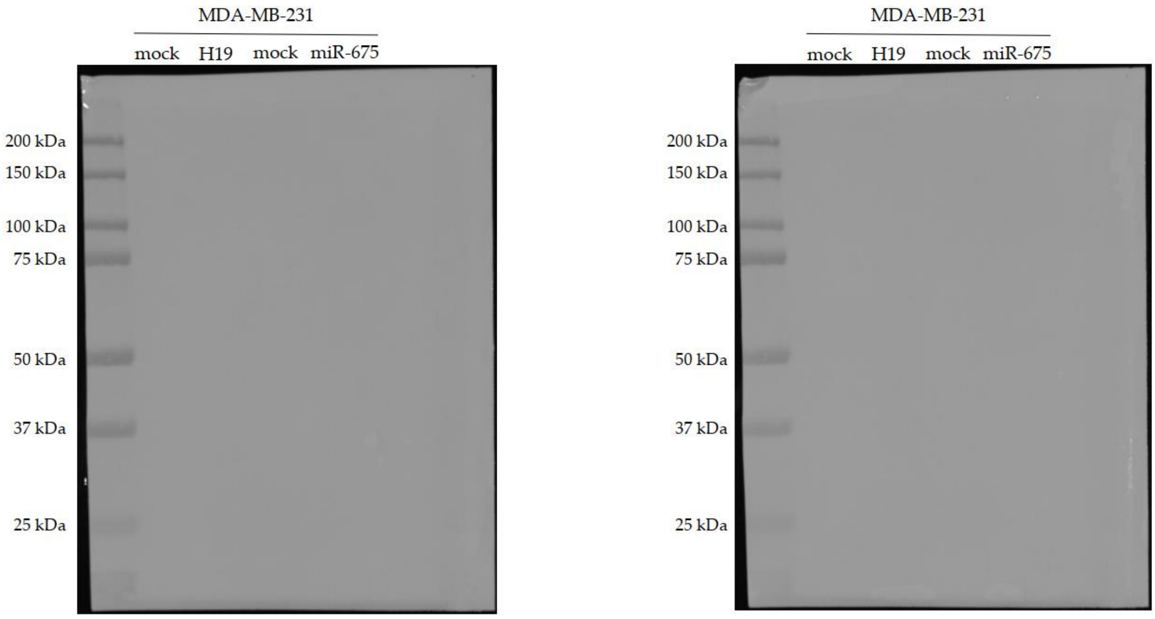

Figure S7. Uncropped western blot membranes (visible condition) corresponding to Figure S8.

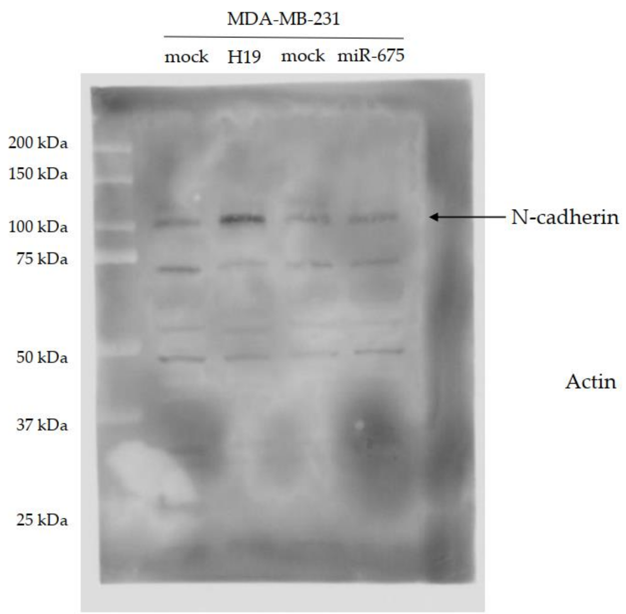

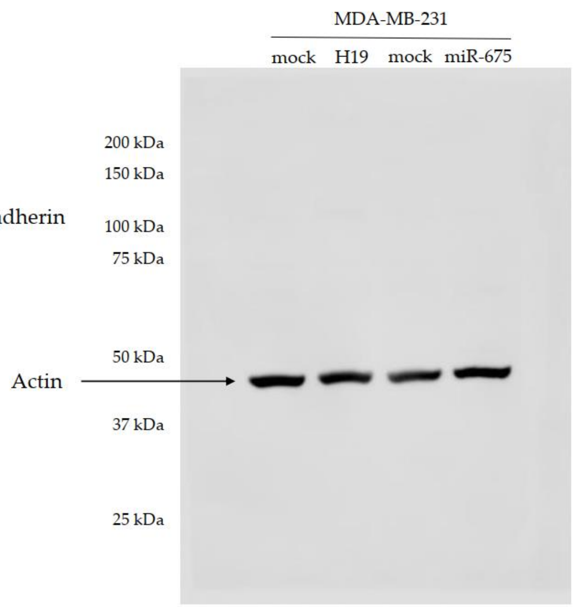

Figure S8. Revelation of uncropped western blot membranes showing the detection of N-cadherin protein expression in MDA-MB-231 control (mock), *H19*-stably overexpressing cells (H19) or miR-675-stably overexpressing cells (miR-675). Actin was used as an equiloading control.

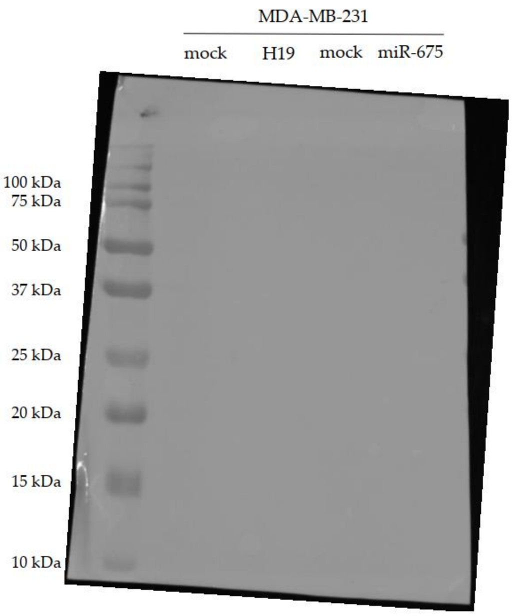

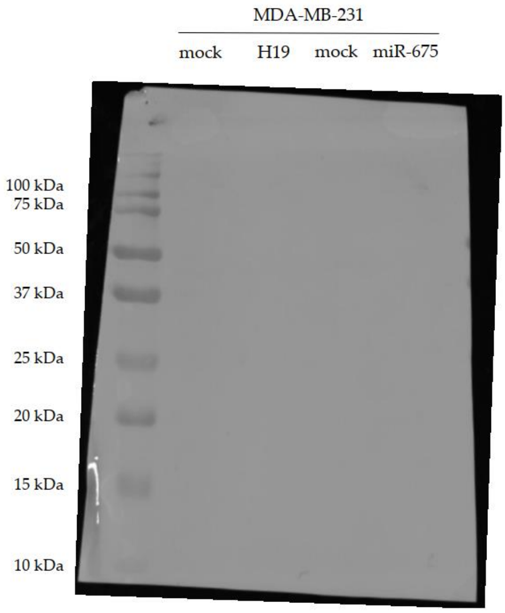

Figure S9. Uncropped western blot membranes (visible condition) corresponding to Figure S10.

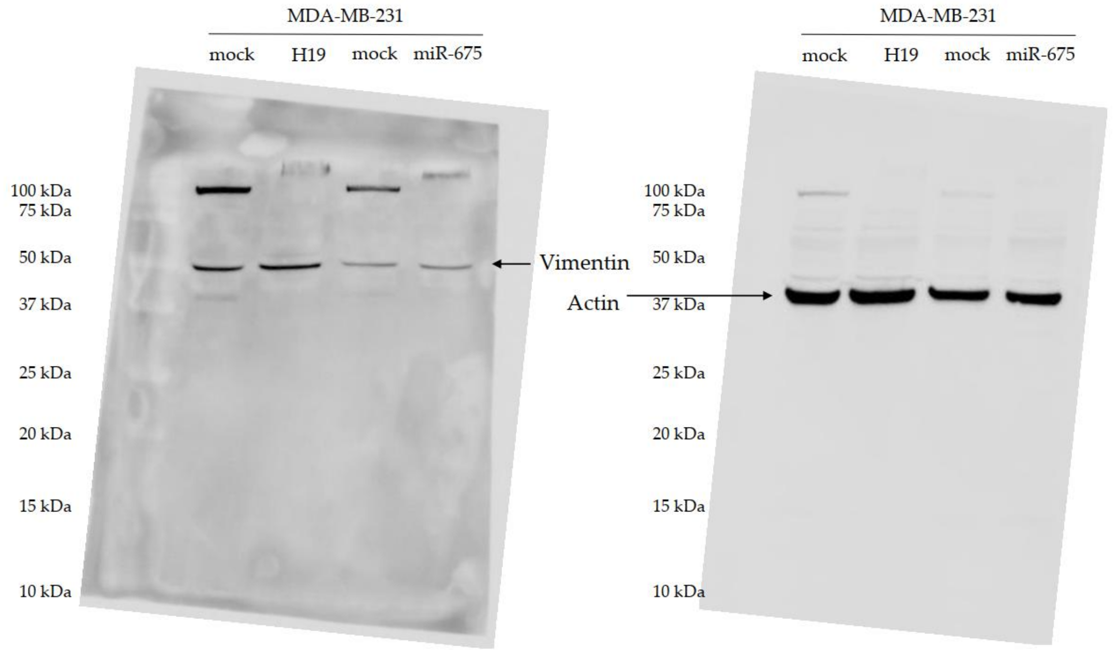

Figure S10. Revelation of uncropped western blot membranes showing the detection of vimentin protein expression in MDA-MB-231 control (mock), *H19*-stably overexpressing cells (H19) or miR-675-stably overexpressing cells (miR-675). Actin was used as an equiloading control.

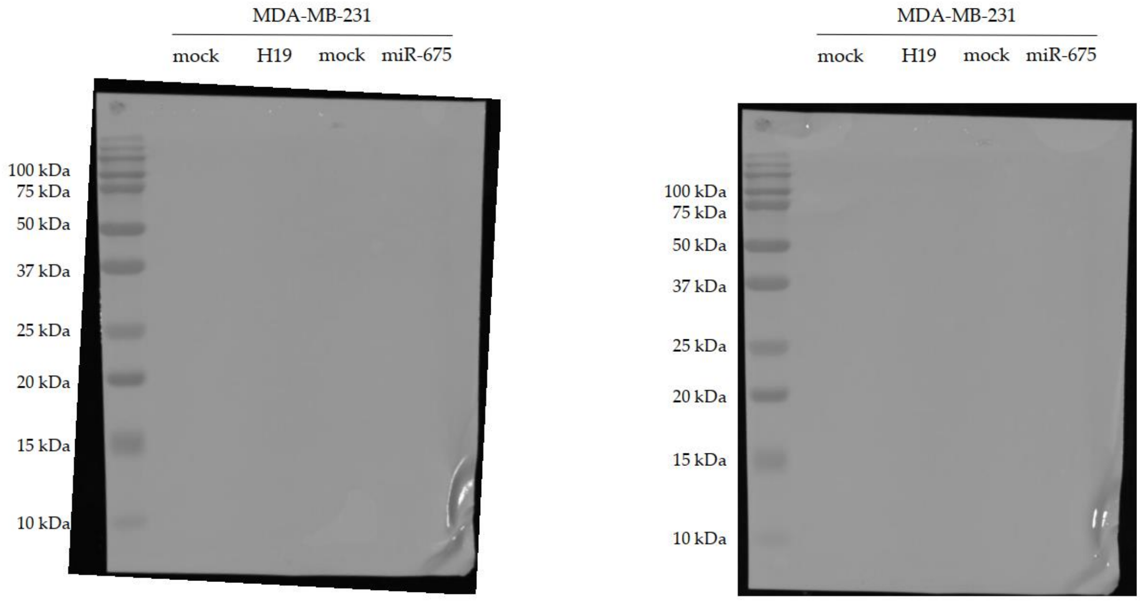

Figure S11. Uncropped western blot membranes (visible condition) corresponding to Figure S12.

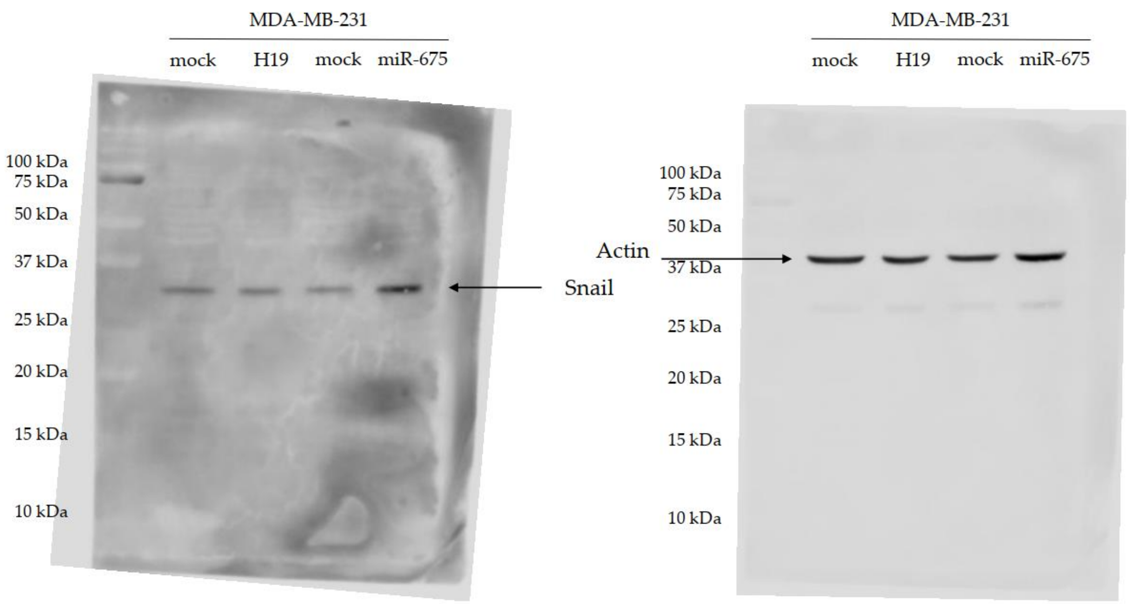

Figure S12. Revelation of uncropped western blot membranes showing the detection of snail protein expression in MDA-MB-231 control (mock), *H19*-stably overexpressing cells (H19) or miR-675-stably overexpressing cells (miR-675). Actin was used as an equiloading control.

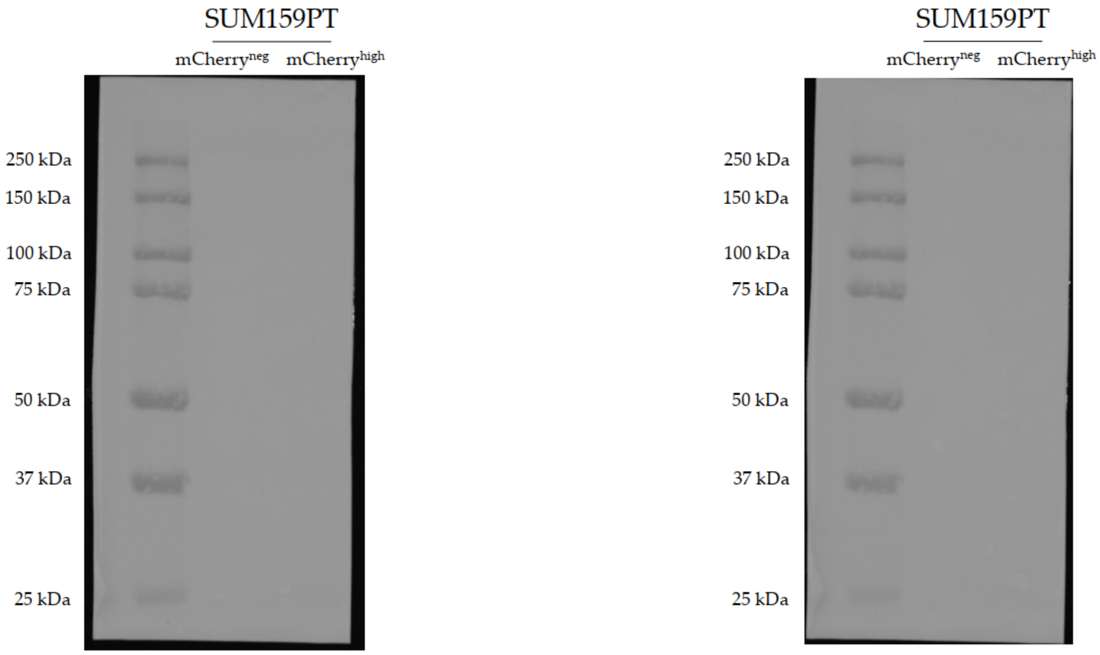

Figure S13. Uncropped western blot membranes (visible condition) corresponding to Figure S14.

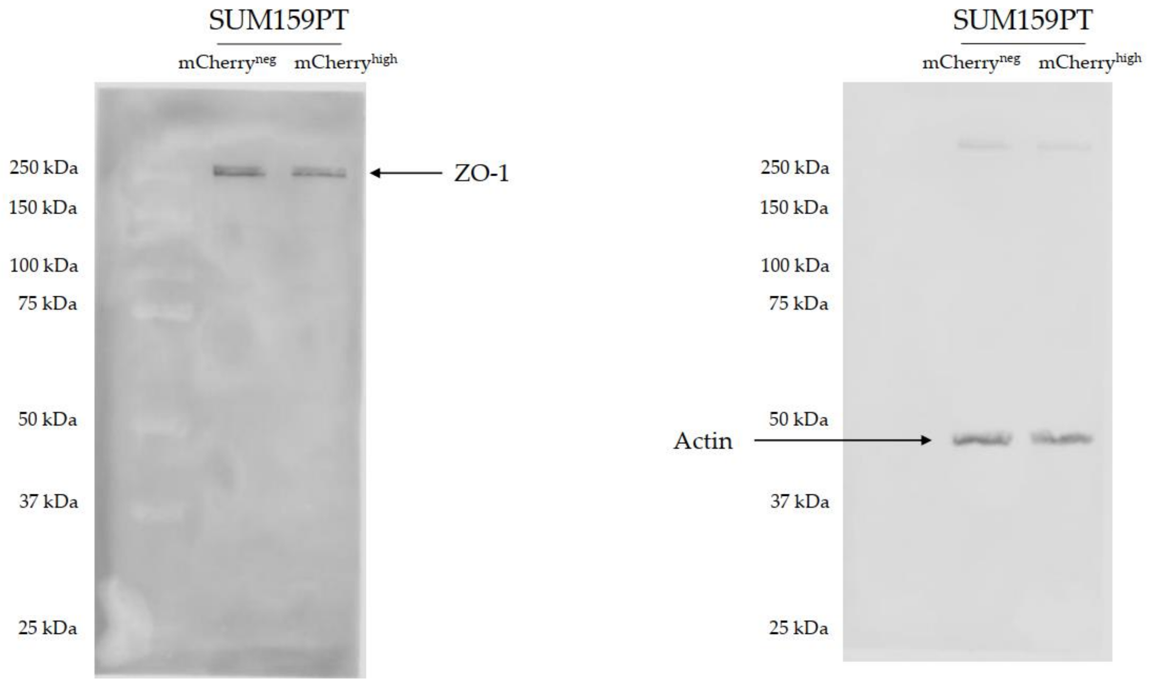

Figure S14. Revelation of uncropped western blot membranes showing the detection of ZO-1 protein expression in SUM159PT-pH19-mCherry^{neg} (mCherry^{neg}) or SUM159PT-pH19-mCherry^{high} (mCherry^{high}) cells. Actin was used as an equiloading control.

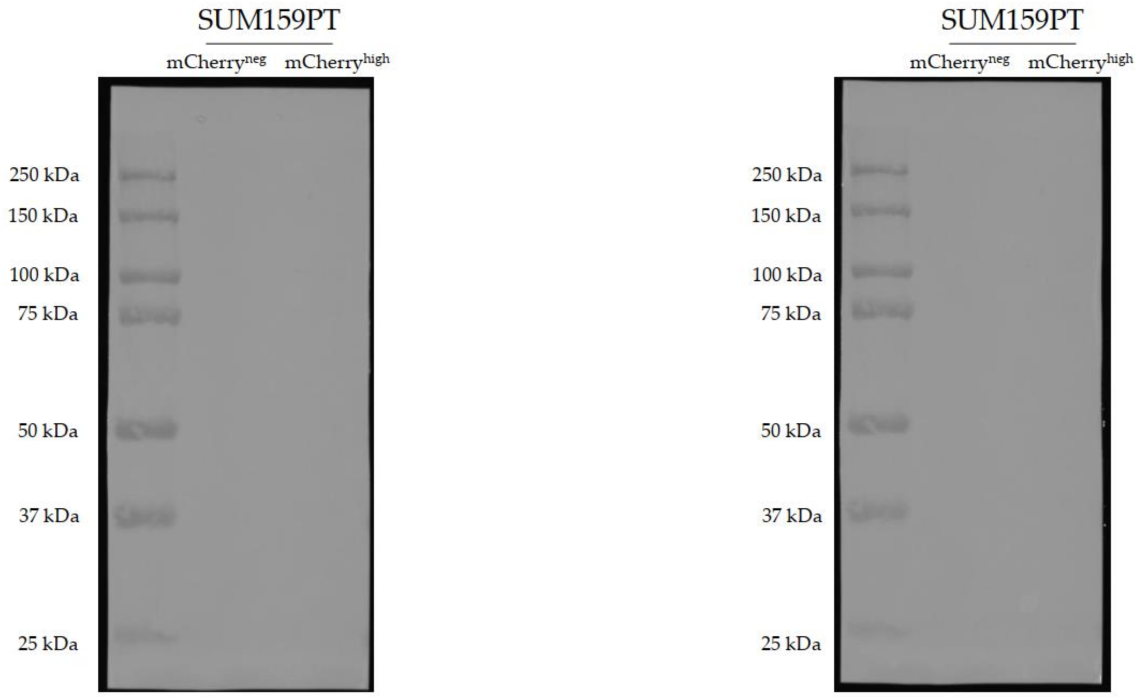

Figure S15. Uncropped western blot membranes (visible condition) corresponding to Figure S16.

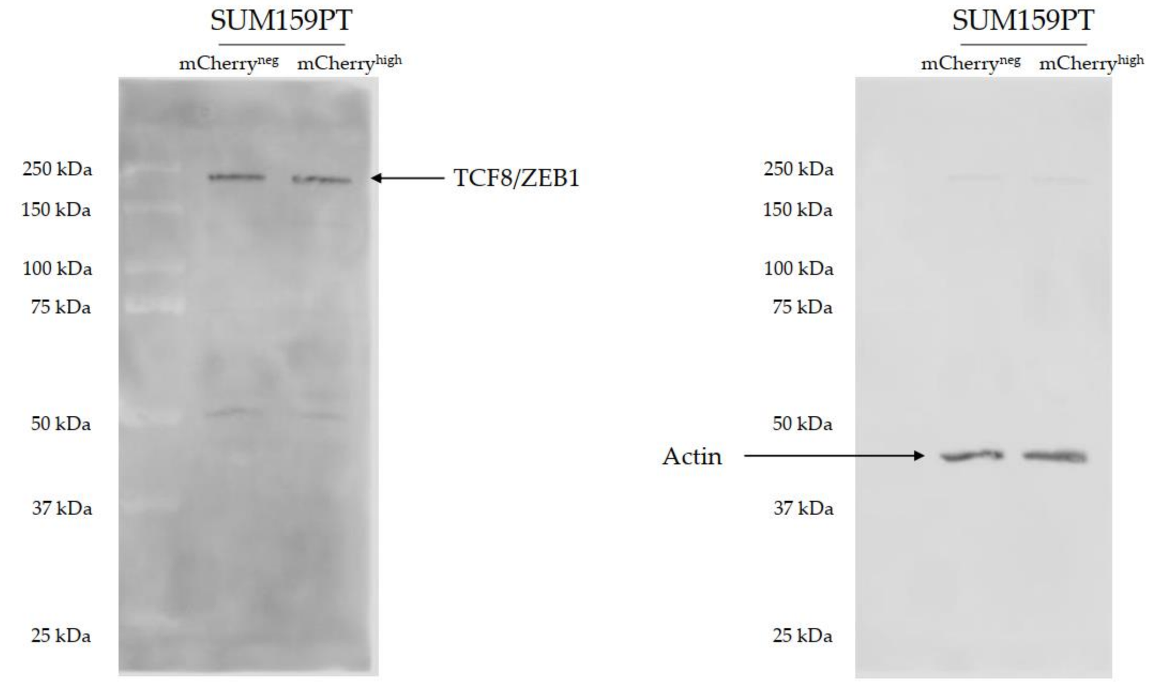

Figure S16. Revelation of uncropped western blot membranes showing the detection of TCF8/ZEB1 protein expression in SUM159PT-pH19-mCherry^{neg} (mCherry^{neg}) or SUM159PT-pH19-mCherry^{high} (mCherry^{high}) cells. Actin was used as an equiloading control.

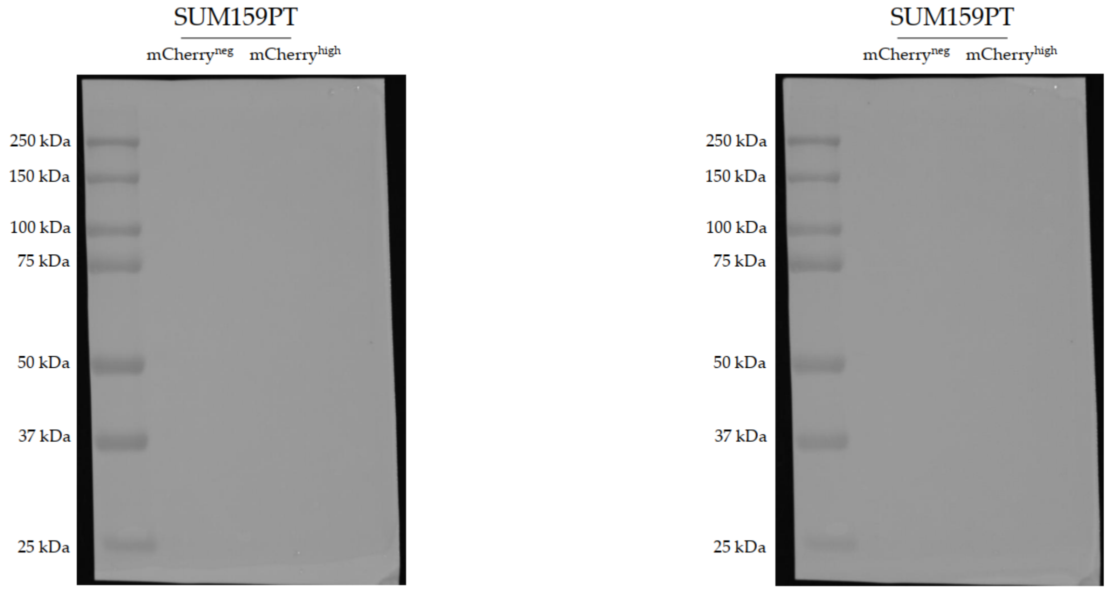

Figure S17. Uncropped western blot membranes (visible condition) corresponding to Figure S18.

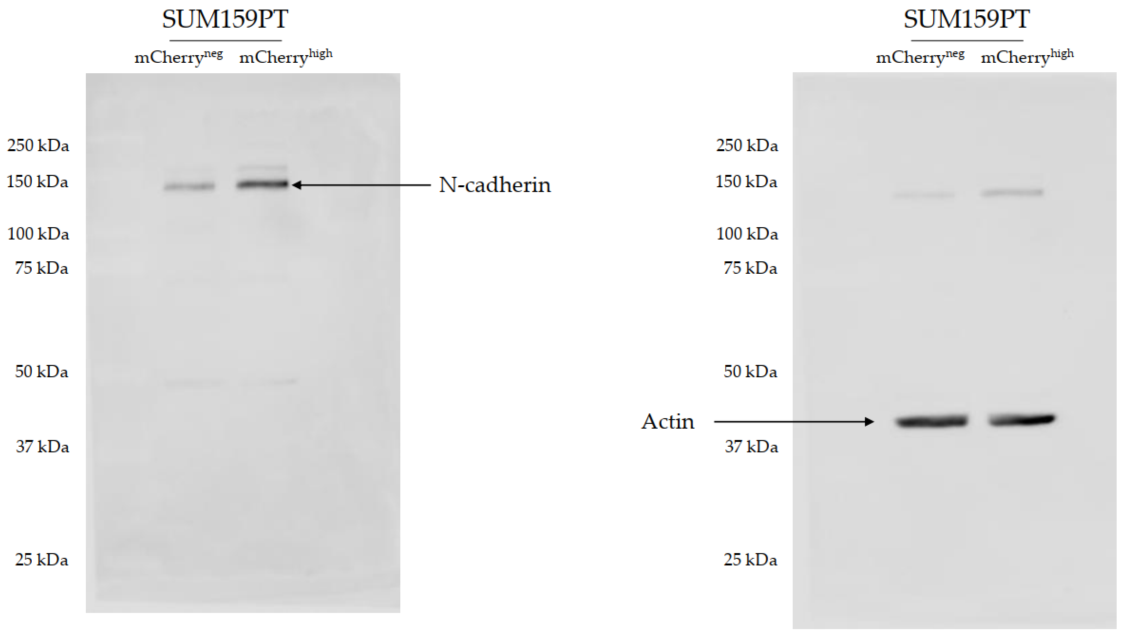

Figure S18. Revelation of uncropped western blot membranes showing the detection of N-cadherin protein expression in SUM159PT-pH19-mCherry^{neg} (mCherry^{neg}) or SUM159PT-pH19-mCherry^{high} (mCherry^{high}) cells. Actin was used as an equiloading control.

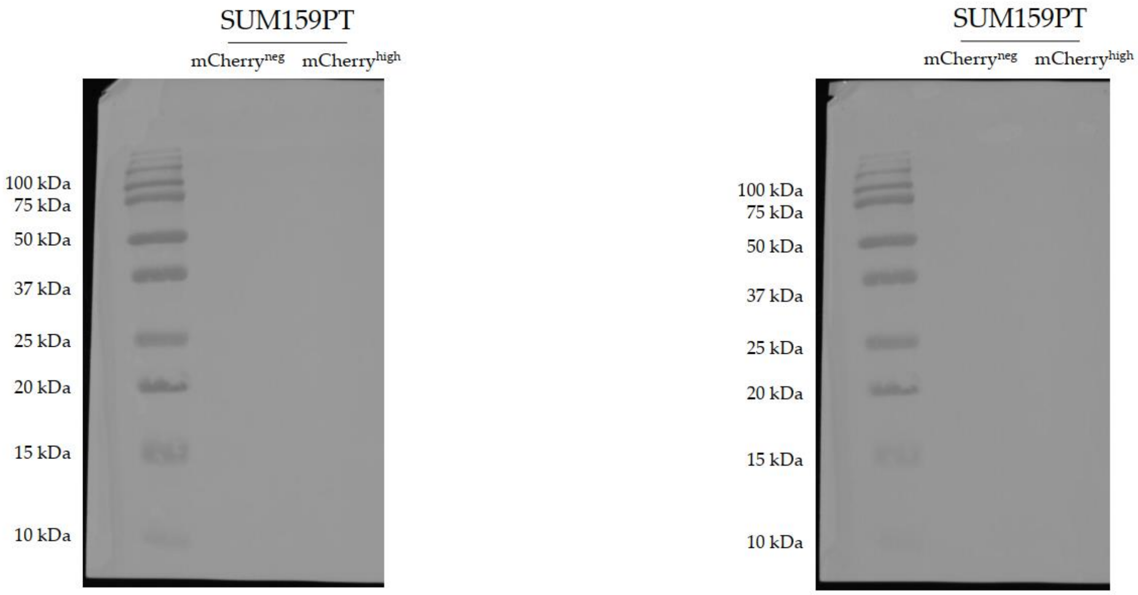

Figure S19. Uncropped western blot membranes (visible condition) corresponding to Figure S20.

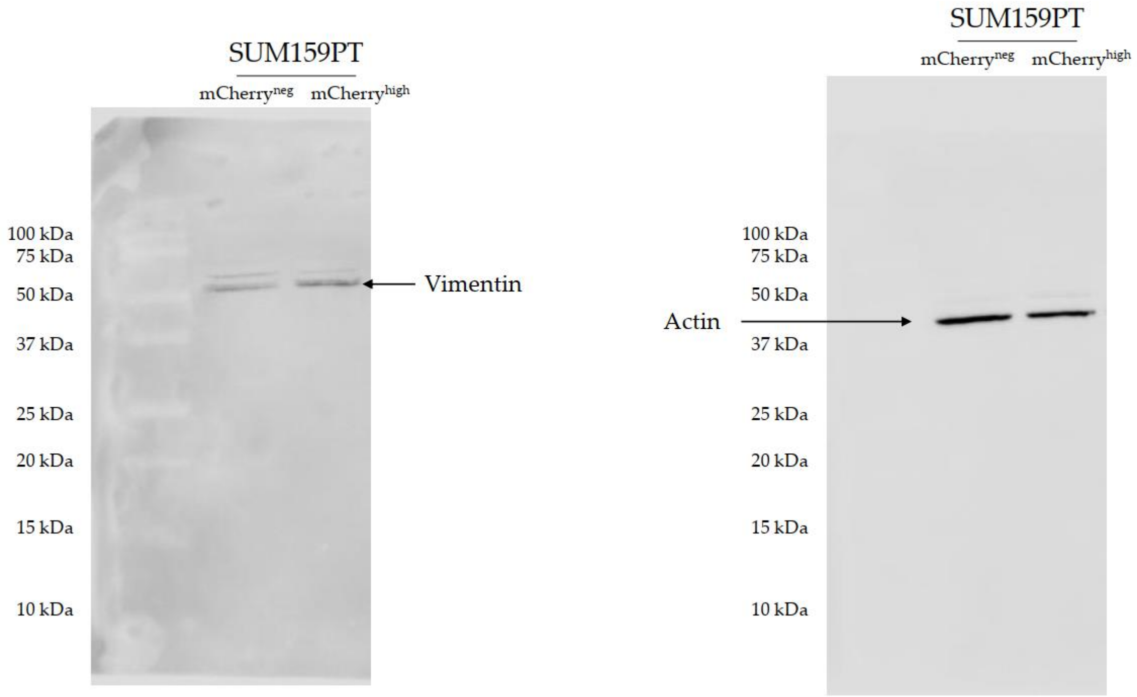

Figure S20. Revelation of uncropped western blot membranes showing the detection of vimentin protein expression in SUM159PT-pH19-mCherry^{neg} (mCherry^{neg}) or SUM159PT-pH19-mCherry^{high} (mCherry^{high}) cells. Actin was used as an equiloading control.

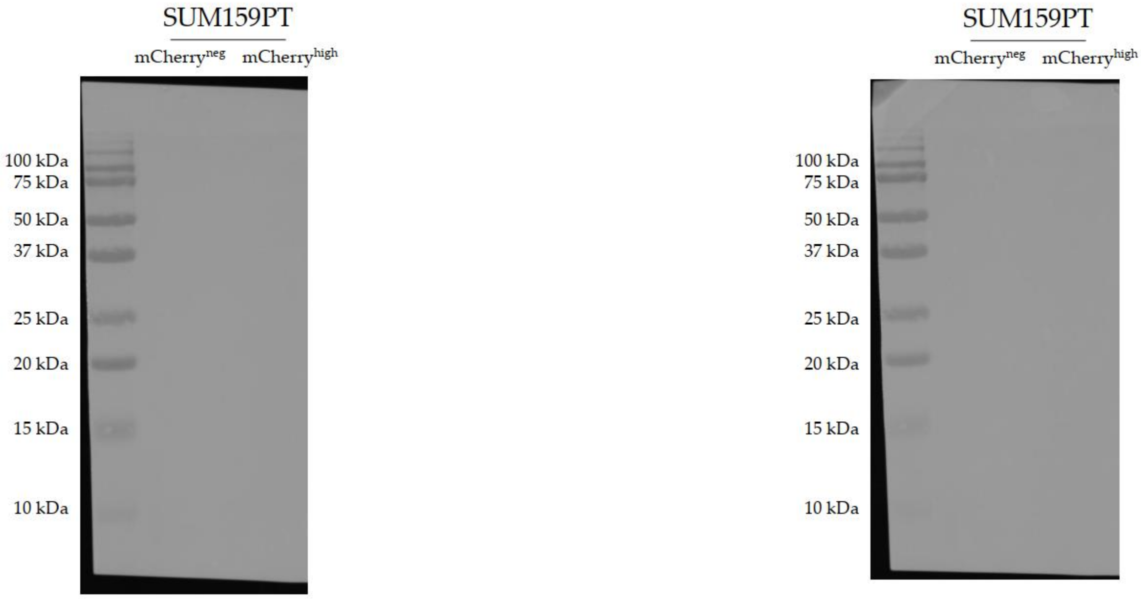

Figure S21. Uncropped western blot membranes (visible condition) corresponding to Figure S22.

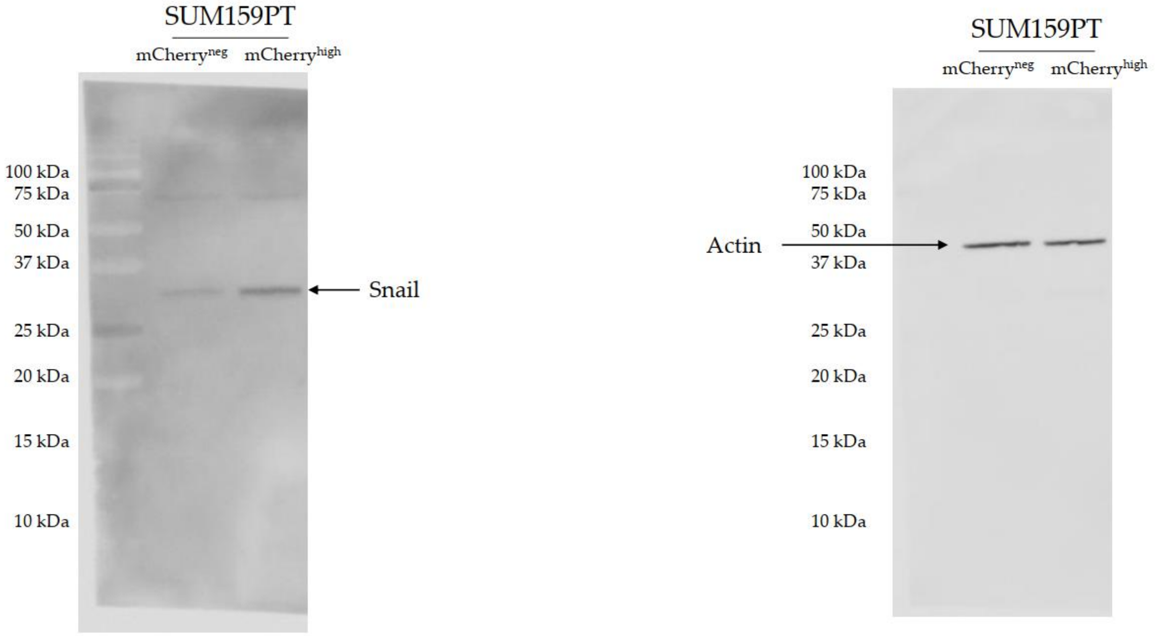

Figure S22. Revelation of uncropped western blot membranes showing the detection of snail protein expression in SUM159PT-pH19-mCherry^{neg} (mCherry^{neg}) or SUM159PT-pH19-mCherry^{high} (mCherry^{high}) cells. Actin was used as an equiloading control.

© 2020 by the authors. Licensee MDPI, Basel, Switzerland. This article is an open access article distributed under the terms and conditions of the Creative Commons Attribution (CC BY) license (http://creativecommons.org/licenses/by/4.0/).
